# Adoption of improved crop varieties limited biodiversity losses, terrestrial carbon emissions, and cropland expansion in the tropics

**DOI:** 10.1073/pnas.2404839122

**Published:** 2025-02-03

**Authors:** Uris Lantz C. Baldos, Alfredo Cisneros-Pineda, Keith O. Fuglie, Thomas W. Hertel

**Affiliations:** ^a^Department of Agricultural Economics, Purdue University, West Lafayette, IN 47907; ^b^United States Department of Agriculture, Economic Research Service, Resource and Rural Economics Division, Washington, DC 20250

**Keywords:** agricultural productivity, biodiversity, land use change emissions

## Abstract

Research investments in crop improvements, including by national and international agricultural research centers, have made significant contributions to raising yields of staple food crops in developing countries. Although mostly intended to improve food security and rural incomes, innovations in crop production also have major implications for the environment. Building on the latest productivity estimates from historical crop improvements in developing countries and using a gridded (0.25 degrees) equilibrium model of global agriculture, we assess the impacts of improved crop varieties on cropland use, threatened biodiversity, and terrestrial carbon stocks over 1961–2015. We replicate a historical baseline and produce a counterfactual scenario which shows the impact of omitting productivity improvements from these technologies. The results show that higher crop productivity generally lowered commodity prices, which reduced incentives to expand cropland except in those areas where productivity gains outweighed price declines. The net global effect of technology adoption was to limit conversion of natural habitat to agricultural use, although it did cause cropland to expand in some areas. We estimate that adoption of improved crop varieties in developing countries saved on net 16.03 [95% CI, 12.33 to 20.89] million hectares worldwide. With more natural habitat preserved, around 1,043 [95% CI, 616 to 1,503] threatened animal and plant species extinctions were avoided over this period. In addition, net land use savings from the improved crop varieties resulted in avoided terrestrial greenhouse gas (GHG) emissions of around 5.35 [95% CI, 3.75 to 7.22] billion metric tons CO_2_ equivalent retained in terrestrial carbon stocks.

## Significance

Agriculture is one of the main drivers of global land use change (LUC) as well as terrestrial carbon and biodiversity losses. While the environmental footprint from agricultural production could be reduced by sustained productivity growth, past studies have ignored local heterogeneity of agricultural production and its impact on biodiversity and terrestrial carbon stocks. Using the latest estimates of productivity impacts from the adoption of improved crop varieties in the developing world and a spatially explicit equilibrium model of global agriculture, our findings show that, at the global level, historical crop improvements over the period 1961–2015 resulted in less cropland expansion, lower LUC greenhouse gas (GHG) emissions and potentially saved thousands of threatened plant and animal species from extinction.

The increase in demand for agricultural commodities due to rising global population and per capita incomes since the mid-20th century have put unprecedented strain on the Earth’s resources. Agriculture currently occupies around 37 percent of the world’s land area (1) and accounts for about one-quarter of global anthropogenic emissions of greenhouse gases (2). Agricultural expansion to meet this growing demand is one of the main drivers of land use change (LUC) and natural habitat loss (2, 3). Over the last two decades, a majority of tropical deforestation can be attributed to agricultural land conversion (4, 5). More broadly, it is likely that most of the net carbon emissions from global land use and land cover change since 1850 are due to agricultural land expansion (6). Changes in agricultural land area also have important implications for biodiversity since LUC is one of the major drivers of global biodiversity loss (7).

Productivity growth offsets the pressure to expand agricultural land area while meeting growing market demand. Early in the 20th century crop yield growth accelerated in many of today’s developed nations; in some developing countries crop yields got a major boost with the onset of the “Green Revolution” in the 1960s. Concerns over burgeoning population and increasing scarcity of land and water resources in the developing world led donor countries and organizations to establish a set of international agricultural research centers in the 1960s to address food crop production constraints in developing countries, which continue to operate today as the Consultative Group for International Agricultural Research (CGIAR).^*^ CGIAR centers working in collaboration with national research institutes have produced a stream of improved crop varieties, initially for wheat and rice and later extended to other food crops, including coarse-grains, root crops, and grain legumes (8). Low and middle-income countries have also increased their investment in agricultural research and development, with these governments more than tripling annual spending since 1981 (9). By 2016–2020, improved varieties of food crops developed from national, CGIAR, and private sector research programs had been adopted on at least 440 million hectares in developing countries, or about two-thirds of the total area sown to these crops (10).

The adoption of higher-yielding varieties and accompanying intensification of crop production in developing countries helped lower food prices and improve global food security (11), but their impact on land use and other environment resources is less clear. If the productivity gains are high enough to offset the effects of lower output prices (and thus raise net returns to farming), then farmers may increase output and expand land in crop production. The net impact of technology adoption on environmental resources depends on the characteristics of land moving into and out of agriculture. Spatial patterns of agricultural LUC are of critical importance for both greenhouse gas (GHG) emissions and biodiversity loss (12).

Using data from Evenson and Gollin (8) on the productivity impacts of adoption of improved varieties for 11 food crops in developing countries over 1965–1998 (and assuming these productivity gains continued through at least 2004), Stevenson et al. (13) estimated that adoption of Green Revolution varieties resulted in a net land savings of 18 to 27 million hectares globally. To derive these estimates, the authors used a global economywide model calibrated for the year 2004 to first determine the impact of the yield improvements attributable to adoption of improved varieties on world commodity prices. They then divided the world into 18 agro-ecological zones (6 each in tropical, subtropical, and temperate environments) and quantified how cropland was affected in each zone in response to these changes in commodity prices. An important advance of ref. 13 over previous work on this topic is that they captured how price changes due to crop productivity improvement affected farmers’ land use decisions, whereas many previous studies relied on simple correlations between yield and area change across countries (14, 15). However, Stevenson et al. (13) were only able to break down land use effects at the highly aggregated agro-ecological zones-regional level, which they showed were globally land-saving due to crop productivity improvements. Their model also assumes that accessible natural land cannot be brought into economic production (i.e., commercial land endowments in each region are fixed) and additional land demand can only be sourced from existing uses (crop, livestock, and managed forest sectors). Finally, the authors only focused on the productivity impacts of Green Revolution technologies on the global economy as it was in the base year, 2004.

Hertel et al. (16), using the same crop productivity estimates as ref. 13, demonstrated that the implications of crop productivity growth on land use are sensitive to how well countries are integrated into global markets, as well as the relative differences among (and within) countries in yields, emissions efficiencies, cropland supply response, and intensification potential. The authors used a global agricultural model which defined agricultural production and cropland supply in 15 world regions. A key contribution of Hertel et al. (16) to the literature is the estimation of a historical baseline which projects the world agricultural sector from 1961 to 2006 using population, per capita income as well as agricultural productivity as key model inputs. This is then compared to a historical counterfactual which omits the productivity gains from Green Revolution varieties. The authors estimated that over 1961–2006 adoption of Green Revolution varieties reduced global cropland expansion by 10% relative to the historical counterfactual. However, the cropland supply parameters in ref. 16 are calibrated at the continental level and do not consider the physical availability of land, potentially leading to overestimation of cropland change. More importantly, both Stevenson et al. (13) and Hertel et al. (16) used models that aggregated crop production into large geographic regions or zones, ignoring local heterogeneity and constraints on agricultural and natural resources, as well as missing the mixed impacts at a finer scale, where cropland in some locations might expand in response to relatively faster rates of productivity growth.

The principal contributions of this study are threefold. First, we disaggregate the impacts of crop productivity growth at the local level by integrating gridded crop production and land use within a market equilibrium model of the global agricultural and food economy (SIMPLE-G) (*SI Appendix*, *Supporting Text*). This enables us to derive estimates of where land use likely changed in response to the diffusion of improved crop varieties, linking these to geospatial changes in terrestrial carbon stocks and biodiversity. Our model tracks crop production and cropland use at 0.25 × 0.25 degree resolution (~27 × 27 km near the equator). We adopt cropland supply functions which incorporate physical limits to available cropland (17, 18) in each grid-cell based on availability of potentially cultivable land (19). Using this model, we assess some of the environmental impacts of the innovations in crop production at fine scale. Following Hertel et al. (16), the experimental design in this study involves simulating a historical baseline (which embeds the productivity impacts resulting from adoption of improved food crop varieties) and a counterfactual scenario (which removes these productivity impacts) for the periods 1961–1985, 1985–2000, and 2000–2015 (*SI Appendix*, Fig. S1). Local changes in cropland use due to improved varieties are then used to calculate terrestrial carbon outcomes. Extending the scope of impacts beyond previous studies, our work also reports changes in biodiversity outcomes using land occupation characterization factors from Chaudhary et al. (20) which represent potential losses in threatened plant and animal species when land is converted to agriculture (*Materials and Methods*).

Second, our study takes advantage of an expanded dataset that adds more crops and two additional decades of data on the diffusion of improved varieties (IV) of food crops in developing countries (10). These authors assembled country-level data on the extent of diffusion of improved varieties for 19 food crops over 1961/65–2016/20 and estimated the crop productivity effects using field-scale data from the literature. These 19 crops account for about two-thirds of the total crop area harvested in developing countries. By 2016/20, the use of improved varieties had spread to more than 60 percent of this crop area and was increasingly geographically dispersed across developing regions (10). After subtracting yield gains due to increased use of fertilizers and other inputs, adoption of improved crop varieties initially raised crop yield by around 30 to 40 percent compared with traditional varieties (with impacts varying by crop and country). Adoption of subsequently improved (new generations of) crop varieties added an additional 10 to 20 percent to crop yield (10). One limitation of ref. 10 is that they only included crops and regions for which CGIAR centers had breeding programs, which restricted the coverage of soybeans to Sub-Saharan Africa. However, soybeans have been expanding rapidly in other tropical regions as well, especially in Brazil where it has been associated with loss of forests and grasslands (5). In an attempt to rectify this gap, we added to ref. 10 information on the impacts of soybean varietal improvement in Brazil (*SI Appendix*, *Supporting Text*). We apply these aggregated crop diffusion rates and yield growth estimates to gridded data on crop production (21) and cropland extent for the year 2015 (22) to determine the productivity improvement in each grid cell and for each period (*SI Appendix*, Fig. S2). Our study estimates not only total impacts over the study period (1961–2015) but also how they evolved over time, and across geographies.

A third contribution of our study is that we isolate the impact of crop varieties developed with germplasm provided by CGIAR centers. CGIAR centers were particularly instrumental in instigating the Green Revolution in developing countries, especially through high-yielding varieties of wheat and rice in Asia and Latin America. Over the last couple of decades, diffusion of CGIAR-related crop innovations has begun to shift geographically, becoming increasingly important in Africa, even as they are gradually replaced by varieties from national programs and the private sector in many other regions. While initially established to address global food security, donor interest in funding the CGIAR has expanded to include natural resource conservation and other concerns. The findings from this study show the extent to which CGIAR research on crop improvement has contributed to these goals.

## Results

### Between 1961 and 2015, Global Cropland Expansion Was Slower Because of Adoption of Improved Crop Varieties.

**Fig. 1.**
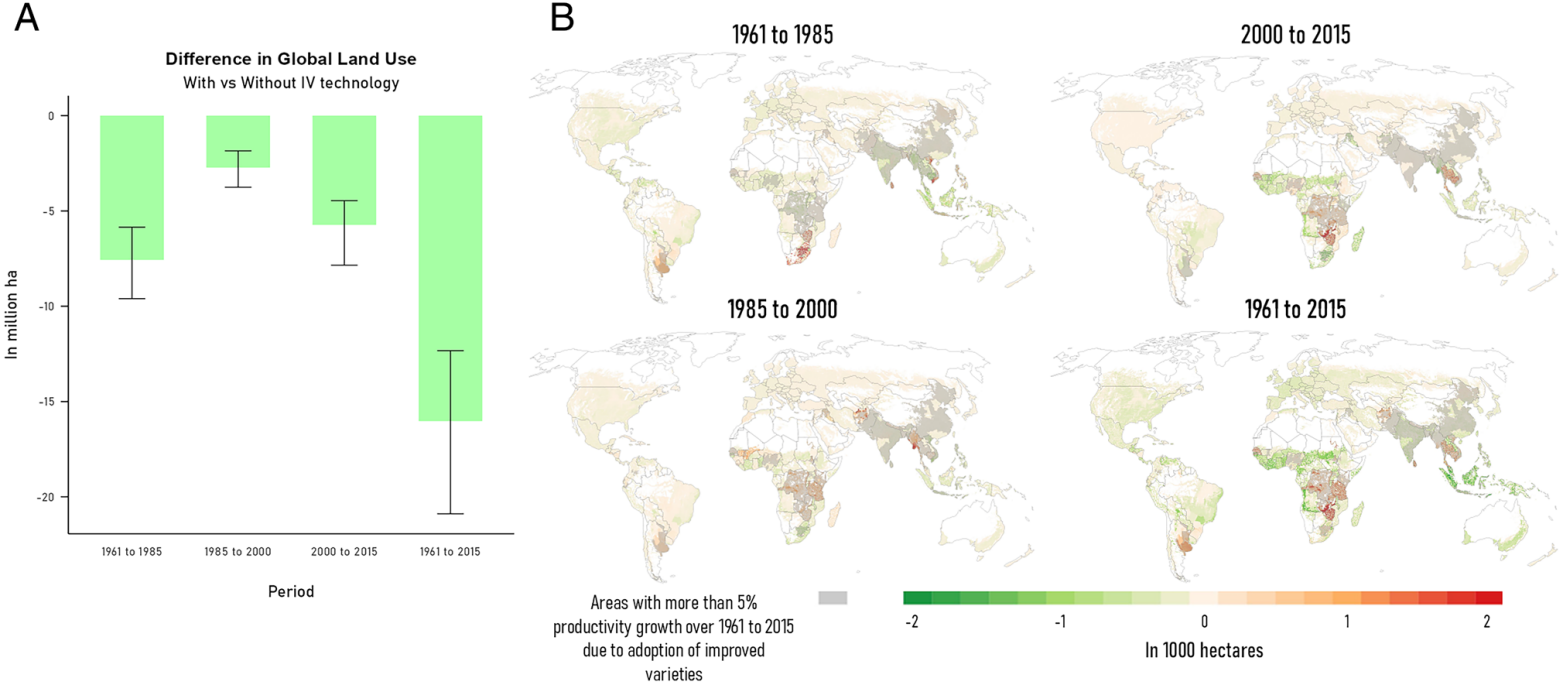
Changes in cropland area due to adoption of improved crop varieties for periods 1961–1985, 1985–2000, 2000–2015, and 1961–2015. (*A*: *Left*) Changes in global cropland area. Error bars show 2.5% and 97.5% percentiles. (*B*: *Right*) Map of average changes in cropland. Green areas are where cropland expansion was lower and red areas are where cropland expansion was higher due to improved crop varieties. Gray shaded areas provide key background information about the extent of adoption, denoting areas with more than 5% additional productivity growth from variety adoption for period 1961–2015.

Additional productivity growth in the developing world from national and international agricultural research resulted in greater crop production and lower cropland use at the global level. From 1961 to 2015, global crop output was higher by 226 million metric tons [95% CI, 212 to 241 million metric tons], calculated as the difference between the baseline and the counterfactual (no improved varieties) while global crop price was lower by −1.72% [95% CI, −1.32% to −2.28%] (*SI Appendix*, Fig. S3). World cropland use was lower by 16.03 million hectares [95% CI, 12.33 to 20.89 million hectares] (Fig. 1*A*) as a consequence of improved crop varieties. This estimate is slightly below the lowest range of avoided land use expansion from Stevenson et al. (13) which used different methods and based on earlier estimates of productivity improvements from improved crops. Fig. 1*A* also shows that more than 47% of these land use savings from improved crop technologies occurred in the 1961–1985 period. After 1985, global cropland use was only reduced by 2.73 million hectares during 1985–2000 and up to 5.73 million hectares during 2000–2015. With the initial switch from traditional to improved varieties, productivity gains during 1961–1985 were generally larger compared to later periods. (*SI Appendix*, Fig. S2).

Fig. 1*B* shows the map of grid-cell level cropland changes from the adoption of improved varieties (IVs) for each period and the cumulative change over 1961–2015. In general, cropland expansion is larger (red areas in Fig. 1*B*) in the presence of the improved technologies in adopting areas (gray shaded areas in Fig. 1*B*) where productivity improvements result in increased profitability. This reflects greater cropland area under the historical baseline scenario (with IV technologies) compared to the counterfactual. In grid cells which are not benefitting directly from these technologies, cropland area under the baseline scenario is much less than under the counterfactual—indicating slower cropland expansion in these areas (green areas in Fig. 1*B*). Looking at the map for the period 1961–2015, most of the IV-induced cropland cover changes occurred in regions which benefited disproportionately from national and international agricultural research such as in the Southern Cone region of South America, as well as in the Eastern region in Sub-Saharan Africa. However, outside these regions, cropland area generally declined in the counterfactual scenario (no IV technologies) relative to the historical baseline. Other regions which experienced additional productivity growth from improved crop variety adoption include South Asia and Southeast Asia. In South Asia, where land availability was more constrained (*SI Appendix*, Fig. S4), adoption of improved variety technologies nonetheless encouraged some cropland expansion in some parts of the region. In Southeast Asia, cropland area increased in regions which benefited from IV crop innovations. Importantly, the land use impacts of IV crop technology adoption in developing countries are transmitted through international trade (via crop lower prices) to Europe, East Asia, Oceania, and North America, resulting in slower cropland use expansion in these regions. Significant variations in outcomes are observed at the local and regional levels which highlights the importance of integrating fine-scale economic modeling within a global agri-food economy model when examining agricultural LUCs and subsequent environmental impacts.

Because technology-driven productivity gains increase economic profitability of crop production in areas which benefited disproportionately from the improved crop varieties, these gains incentivize further cropland expansion This is termed Jevons paradox, because becoming more efficient increases competitiveness, thereby encouraging area expansion (23, 24). However, as illustrated in Fig. 1*B*, focusing only on those areas which benefitted the most from improved crop variety adoption gives an incomplete picture. Through the resulting drop in commodity prices, cropland area other areas decline and this decline can outweigh the cropland expansion at larger scales.

When cropland changes are aggregated to the regional level, beneficiaries of IV technologies showed less cropland expansion under the historical baseline compared to the counterfactual without IV technologies (*SI Appendix*, Fig. S5). For example, net cropland use in Sub-Saharan Africa was lower by around 2.73 million hectares [95% CI, 1.85 to 4.65 million hectares], as a result of the improved varieties, over the period 1961–2015. Under the historical baseline, regional cropland area in South Asia and in Southeast Asia was also smaller than in the counterfactual (no IV) scenario. Cropland also contracts in regions which are not direct recipients of IV technologies such as North America and Europe. These regional outcomes provide support for the “Borlaug Hypothesis” which argues that technologies that boost agricultural productivity could prevent cropland expansion and deforestation (25, 26). These results are consistent with previous studies which showed that the historical Green Revolution moderated large-scale, regional land use and likely reduced cropland use at the global level (11, 13, 16).

### Reduction in Cropland Expansion Due to Adoption of Improved Crop Varieties Saved Vulnerable Animal and Plant Species from Extinction.

**Fig. 2.**
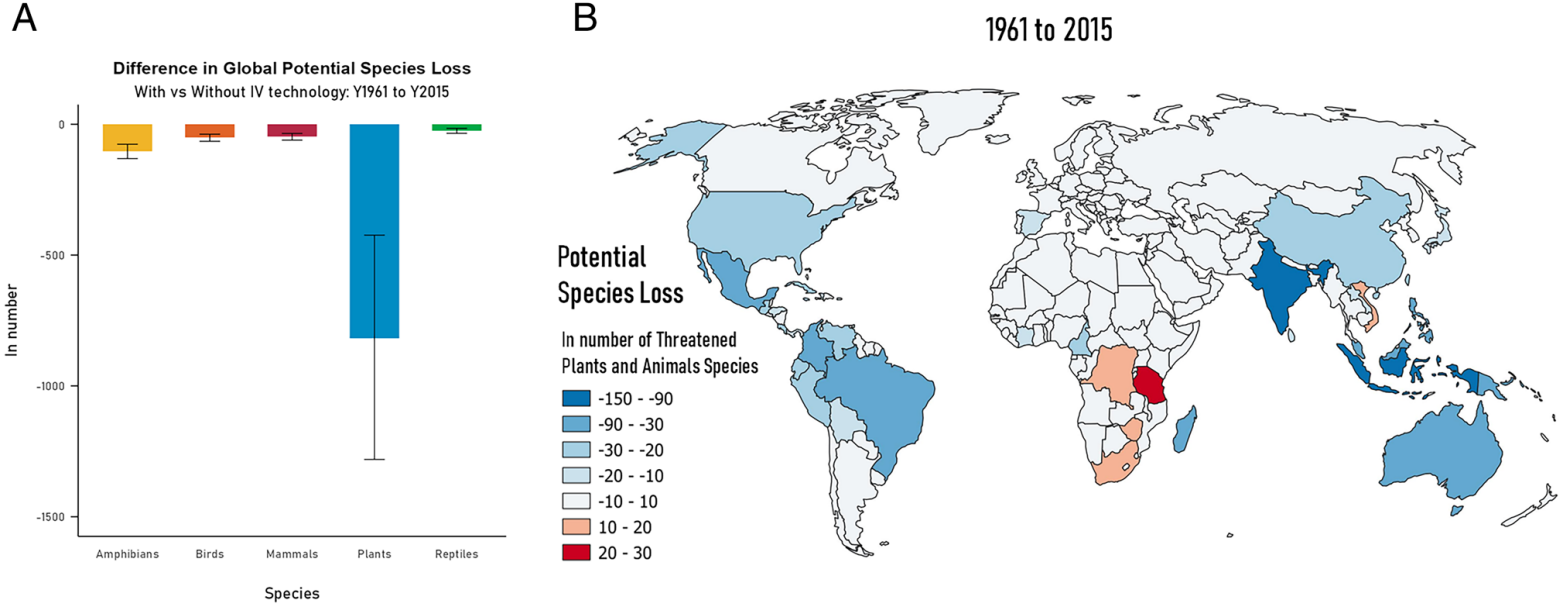
Changes in potential species loss due to adoption of improved crop varieties for period 1961–2015. (*A*: *Left*) Global changes in potential species loss by species type. Error bars show 2.5% and 97.5% percentiles. (*B*: *Right*) Map of average changes in potential species loss for both plants and animals by country. Blue areas are where potential species loss was lower due to varietal adoption. Red areas are where potential species loss was higher due to varietal adoption.

Slower cropland expansion from farmer adoption of IV-related crop innovations resulted in more natural habitats and fewer biodiversity losses than would have occurred in its absence. Based on our analysis, around 1,043 threatened animal and plant species [95% CI, 616 to 1,503] globally were saved due to slower cropland expansion under the historical baseline with improved crop varieties relative to the counterfactual scenario over the period 1961–2015. More than half of the avoided animal and plant species losses occurred during the period 1961–1985 (517 [95% CI, 304 to 756 thousand] *SI Appendix*, Fig. S6). Fig. 2*A* breaks down the number of species which avoided extinction as a result of these agricultural technologies. Around 818 plant species were saved over the period 1961–2015 [95% CI, 424 to 1,281]. There are fewer number of animals species saved compared to plants species. Average avoided losses in animal species include 103 amphibians [95% CI, 76 to 131], 50 birds [95% CI, 38 to 65], 47 mammals [95% CI, 35 to 61], and 25 reptiles [95% CI, 16 to 35]. It should be noted that there are large uncertainties with respect to the characterization factors, especially for plant species (*SI Appendix*, Table S2), which is indicative of the difficulty in measuring biodiversity impacts from LUC.

Fig. 2*B* shows the global country map of the change in potential species loss, both for animals and plants, between the historical baseline and the counterfactual scenario over 1961–2015. The map shows that there were more countries where species were conserved (the blue areas in Fig. 2*B*) than where species were likely lost (the red areas in Fig. 2*B*). Biodiversity losses as a result of adoption of the improved crop varieties are observed in parts of Sub-Saharan Africa and Vietnam. Recall that these are generally the same regions that benefited disproportionately from these new technologies and experienced greater cropland expansion as a result of increased profitability (*SI Appendix*, Fig. S2). In Brazil, Indonesia, and India, there is biodiversity conservation (or a slowdown in rate of biodiversity loss) despite adoption of improved crop varieties. In general, regions which benefitted relatively little from improved crop varieties yet faced lower crop prices (which reduced incentives to expand cropland) experienced a reduction in the rate of species loss. Moreover, the impacts of IV crop technology adoption in developing countries are transmitted through international trade (via lower crop prices), resulting in land-based biodiversity conservation in East Asia, Oceania, and North America.

Fine-scale modeling of global crop production allows us to identify where avoided extinction occurs and if these are in areas where biodiversity is threatened. We use the biodiversity hotspots (27, 28) to estimate the number of threatened plant and animal species which avoided extinction in these sensitive areas (*SI Appendix*, Fig. S7). These hotspots are regions which have at least 1,500 plants as endemic species (>0.5% of the world’s total) and have 30% or less of original vegetation remaining. They are also home to many endemic plants and terrestrial vertebrate species (27, 29). We find that roughly 80% of the avoided losses in plant species are located within 31 out of 34 biodiversity hotspots which are mapped in our model (*SI Appendix*, Fig. S8) indicating that crop productivity growth from IV technologies generally resulted in conservation of threatened plant and animal species in most biodiversity hotspots.

### Slower Cropland Expansion Due to Adoption of Improved Crop Varieties Reduced Terrestrial GHG Emissions from LUC.

**Fig. 3.**
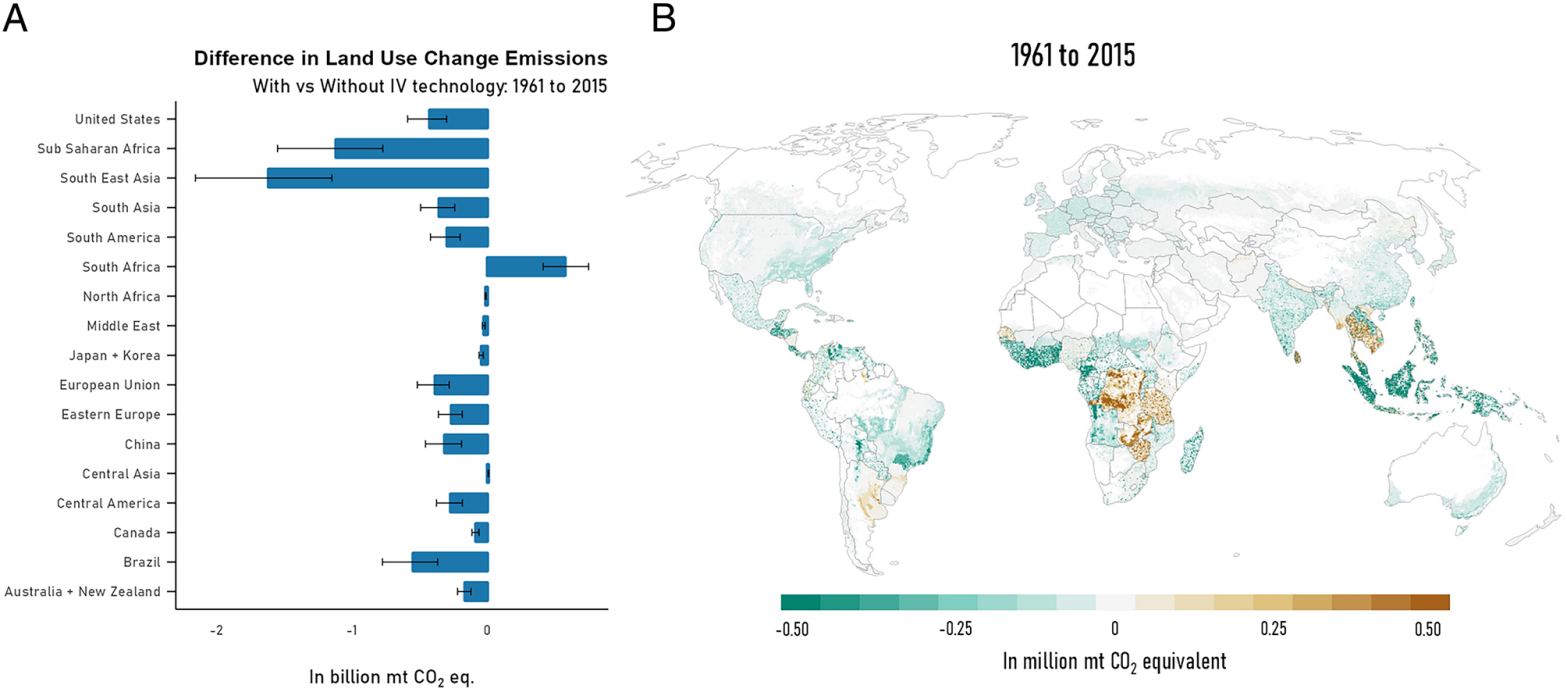
Changes in LUC GHG emissions due to adoption of improved crop varieties for period 1961–2015. (*A*: *Left*) LUC emissions by region. Error bars show 2.5% and 97.5% percentiles. (*B*: *Right*) Map of average changes in LUC emissions. Green areas are where LUC emissions were lower and brown areas are where emissions were higher due to varietal adoption.

The rate at which improved crop variety adoption led to avoided terrestrial carbon emissions from global LUC fluctuated over time: from 3.04 [95% CI, 2.19 to 4.10], to 0.81 [95% CI, 0.52 to 1.15] and then 1.50 [95% CI, 1.04 to 2.04] billion metric tons CO_2_ equivalent over the periods 1961–1985, 1985–2000, and 2000–2015, respectively. In total, global LUC emissions under the historical baseline are lower by 5.35 [95% CI, 3.75 to 7.22] billion metric tons of CO_2_ equivalent compared to the counterfactual scenario without improved crop technologies. Despite lower global land use savings in this study, these GHG estimates are close to the mean estimates in ref. 13 which used regional crop LUCs and regional average carbon emissions data. This highlights the importance of using global gridded modeling which takes full advantage of high-resolution data on terrestrial carbon emissions from agriculture. Fig. 3*A* shows the regional changes in LUC GHG over the period 1961–2015. Most of the avoided land use emissions are in Southeast Asia [1.6 billion metric tons of CO_2_ equivalent, 95% CI 1.1 to 2.2] and Sub-Saharan Africa [1.1 billion metric tons of CO_2_ equivalent, 95% CI 0.8 to 1.5]: These are generally located in tropical forest biomes which have high terrestrial carbon and are rich in biodiversity. Although its regional share of avoided LUC, as measured in hectares, was just 11%, Southeast Asia accounted for about 28% of total avoided LUC GHG emissions. This is due to the fact that the avoided LUC in this region was located in carbon-rich tropical forests where the carbon stock per hectare is very high. Fig. 3*B* shows the map of avoided GHG LUC emissions over the period 1961–2015. Some areas show greater LUC emissions due to additional cropland expansion from IV technology. These are located in the Southern Cone region in South America, parts of South East Asia as well as in the Eastern region in Sub-Saharan Africa.

### CGIAR Technologies Contributed Greatly to Boosting Crop Production and Reducing Agriculture’s Environmental Footprint.

**Fig. 4.**
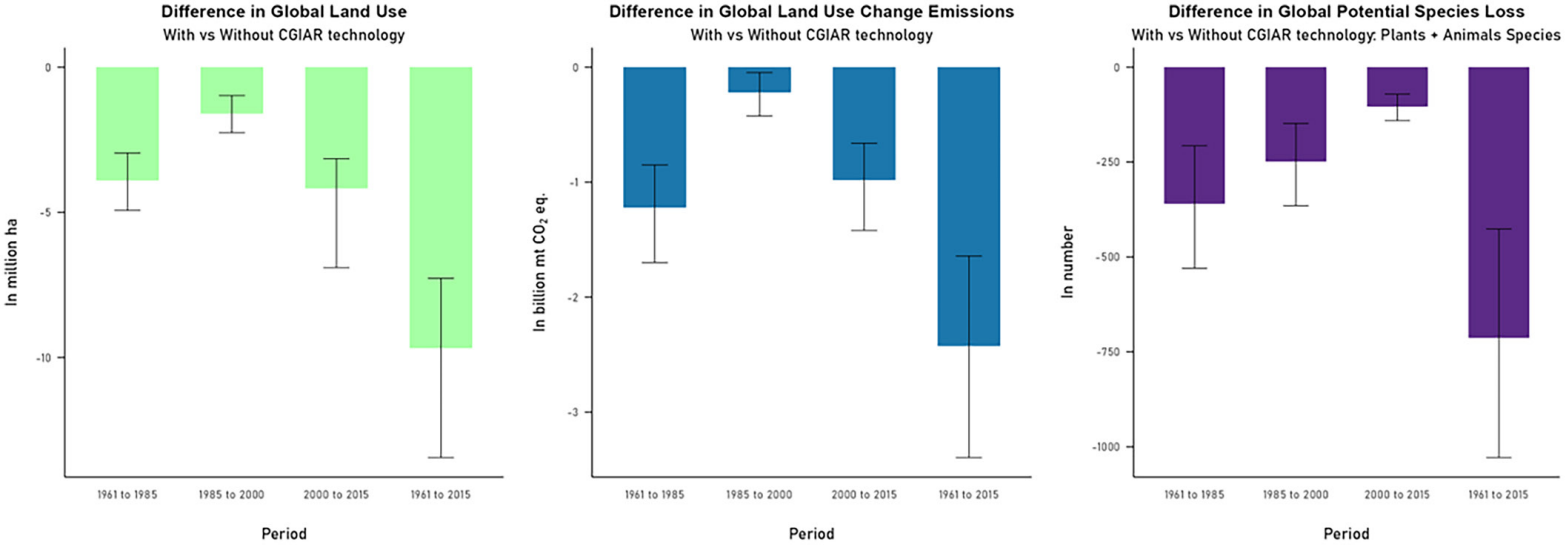
Global changes in cropland use change, LUC GHG emissions, and potential species loss due to adoption of CGIAR varieties only for period 1961–2015. Error bars show 2.5% and 97.5% percentiles.

We isolated the impacts of crop varieties developed with germplasm provided by CGIAR centers over the period 1961–2015 (Fig. 4 and *SI Appendix*, Fig. S9). Because it only focuses on a subset of all improved crop varieties, the productivity growth from CG technologies over this period are relatively smaller and less widespread compared to all improved varieties. Globally, CGIAR technologies contributed roughly 47% of the total productivity gains from adoption of improved crop varieties in developing countries (10). From 1961 to 2015, global crop output was higher by 100 million metric tons [95% CI, 95 to 106 million metric tons in corn equivalent tons] given CGIAR technologies only. This is around 44% of the increase in global crop production when all improved crop varieties are considered. With greater world production, these technologies lowered global crop price by around −1.11% [95% CI, −0.96% to −1.34%]. Adoption of CGIAR technologies had a notable impact in reducing cropland use and subsequent LUC GHG emissions and biodiversity loss. World cropland use was reduced by 9.68 million hectares [95% CI, 7.27 to 13.44 million hectares] over the period 1961–2015 which is 60% of the global cropland use reduction when IV technologies are adopted. Global LUC emissions are also lower by 2.42 [95% CI, 1.64 to 3.39] billion metric tons of CO_2_ equivalent and around 713 [95% CI, 427 to 1,028] vulnerable plant and animal species avoided extinction. Productivity gains from CGIAR technologies are relatively smaller in Argentina, Brazil, China, South Asia, and Southeast Asia (*SI Appendix*, Fig. S9) compared to the gains from all IV technologies (*SI Appendix*, Fig. S2). For these regions, the share of improved varieties planted with CGIAR crop varieties generally started to decline by the 1990s and were subsequently replaced by varieties developed by national research programs or the private sector (10).

## Discussion

In this study, we estimated the terrestrial carbon and biodiversity impacts of adopting improved crop varieties in developing countries using the latest estimates of these productivity gains and a newly developed, spatially explicit global model of agriculture. We find that, at the global level, the diffusion of improved crop technologies dampened cropland expansion, thereby preserving natural habitat, land-based carbon stock, and biodiversity. Overall, cropland savings outweighed cropland expansion for a net savings of 16.03 million hectares, on average, with a 95% CI at around 12.33 to 20.89 million hectares worldwide. This range overlaps with the range from Stevenson et al. (13) which used different methods and based on earlier estimates of productivity improvements from improved crops. Most of the natural lands which were saved from cropland expansion tended to be rich in biodiversity and terrestrial carbon, and as a consequence improved crop innovations reduced potential species loss and terrestrial GHG emissions. However, the results at the grid-level are more nuanced. In innovating areas, which benefited the most from improved crop technologies, crop profitability increased, leading to greater returns to cropland and further expansion. However, in other locations, market-mediated effects via international trade (i.e., lower crop prices) lowered returns to land, which slowed cropland expansion in these regions. Our work highlights the importance of using grid-level analysis since regional-level models ignore spatial heterogeneity of biodiversity and terrestrial impacts which could lead to the underestimation of avoided species loss and GHG emission savings from IV technology adoption (*SI Appendix*, *Supporting Text*).

Our results provide additional evidence for the land-sparing approach to nature conservation which argues that more land could be available for natural uses if agricultural productivity increases in intensive regions (7). The land-sparing effects from technological improvement are stronger in locations where cropland supply is more constrained (14, 30) and food demand is less responsive to prices (30, 31). In the case of the CGIAR, crop research was targeted toward staple crops which are extensively grown and tend to be price-inelastic in demand (13, 31). Of course, environmental outcomes could be further enhanced if efforts to increase agricultural productivity are coupled with direct conservation policies which restrict land use in environmentally sensitive areas (13, 32). Combining both policies is particularly relevant for Sub-Saharan Africa where only 33% of cropland area was planted with improved varieties in 2016–2020. This is particularly important, since agriculture is likely to expand further in Sub-Saharan Africa as population is expected to reach nearly 2 billion people by midcentury (33), and given the region’s vast areas of land which are potentially available for agriculture (19).

The potential gains from the adoption of improved varieties which were estimated in this study are likely conservative since we are only evaluating these over the period 1961–2015. Productivity impacts of improved crop varieties typically span several years and could go well beyond the period examined in this study. Some widely adopted varieties for key crops in Asia and Africa have a slow turnover rate with average varietal age at around 20 y (10, 34). Further, in measuring the terrestrial carbon benefits, we are only focusing on the immediate changes in the above- and below-ground carbon stocks, ignoring carbon flows that occur over time. This is particularly relevant for avoided conversion of young forests which have greater potential to store carbon over time. The impact of natural habitat conversion on threatened species could also extend beyond the period when land is used in agriculture. The regeneration of species population after agricultural land reverts back to its natural state (i.e., land transformation factors) could extend to hundreds of years (20, 35, 36) and factoring this into our analysis would result in significantly larger estimates of threatened plant and animal species avoiding extinction due to slower cropland expansion.

Though our modeling approach offers a significant advance, data limitations and uncertainties surrounding model parameters provide ample opportunities for further research on these issues. One limitation concerns the estimates of the extent of diffusion and productivity impact of CGIAR technologies. As discussed in (10), data on technology adoption and diffusion are measured differently across studies and are not always reliable. Empirical estimates of productivity gains in the literature which use farm-level data could also be biased due to the difficulty in measuring the productivity effects between adopters and nonadopters. Another limitation is the challenge of determining the precise location, timing, as well as where and when these innovations were adopted. We assume these technologies were uniformly adopted in the countries where these crops are grown based on available crop production maps (21). However, these technologies may have favored (initially at least) certain areas over others, such as irrigated cropland or farms that were more closely connected to markets. Potential species loss due to intensive fertilizer and pesticide use are also not measured in this study. We also exclude feedback effects between preservation of natural habitats and agriculture, such as crop pollination, which could be enhanced due to proximity to natural lands.

An additional set of limitations stems from our modeling framework which does not fully capture key mechanisms and drivers of grid-level crop production and cropland use at the local level. Our model assumes that crop producers at the grid level are well integrated into the regional crop market, and they can readily adjust their output and input use in response to changes in market prices. However, factors such as distance to markets, communication networks, and transportation infrastructure can have significant impacts on how farmers respond to prevailing market prices (37–39). Market-driven conversion of available land into crop production might not be possible in some areas due to lack of formal property rights for land (40, 41), increases in protected land in environmentally sensitive areas (42) or shifts in government land use policies (43). Another limitation of our model is that crop demand is still determined at the regional level and not at the local level. Future work should focus on incorporating more realistic assumptions on how farm and nonfarm households interact at the grid-cell level to help improve the accuracy of these estimates. There are also significant uncertainties in the gridded data on nonland and land supply elasticities as well as GHG emissions and biodiversity losses from LUC. Through sensitivity analysis, we have explored different assumptions about key parameter values to establish upper and lower bounds around our estimates. However, more precise measures of these factors will improve our understanding of the relationship between agricultural productivity and the use of these environmental resources.

Last, our study does not take into account the full set of environmental and health consequences that may accompany crop intensification resulting from adoption of improved crop varieties. Pingali (44) reviews evidence that adoption of high-yielding crop varieties sometimes led to overuse of fertilizers, pesticides, and irrigation water with negative consequences for natural resources and human health. In a careful study on the environmental and health consequences of pesticide use in Philippine rice production following adoption of improved varieties, for example, Pingali and Roger (45) found significant negative impacts on farmers’ health due to pesticide exposure. Another example comes from the South Asia Punjab, where Mugai et al. (46) found evidence of long-term soil degradation (declining soil organic matter and tubewell water quality) following the intensification of agricultural productivity instigated by the Green Revolution. Often these impacts are highly local, whereas our focus on LUC, GHG emissions, and biodiversity has global as well as local consequences.

The agricultural model that we developed in this study incorporates global macroeconomic drivers of food demand as well as international trade, in addition to defining crop production and cropland supply at the grid-level. The grid-level resolution of our model allows us to capture dynamics of cropland change at the local level and readily incorporate spatially explicit data on cropland availability, terrestrial carbon stock, and biodiversity outcomes. Despite its limitations, our work is an improvement over previous assessments regarding the historical impacts of improved variety adoption which relied on models which are highly aggregated and fail to account for cropland availability as well as agriculture’s environmental footprint at the local level. Given the flexibility of our modeling framework, it can be used to help inform policymakers on the potential trade-off and synergies of global conservation efforts which target areas with high concentrations of biodiversity and terrestrial carbon [e.g., the “30 × 30” Target in the Global Biodiversity Framework (47)]. It can also be coupled with biophysical models of land use, biodiversity, and ecosystem services to examine local to global sustainability issues in light of future trends in productivity and macroeconomic drivers of global agriculture.

## Conclusions

This study takes advantage of a comprehensive dataset of historical estimates of productivity gains from improved variety adoption in the developing world. In addition, this study examines the terrestrial carbon and biodiversity impacts of such innovations at the level of individual grid cells using a spatially explicit model of agriculture which we developed for this work. We begin by building a historical baseline in which we simulate the development of global agriculture over the period 1961–2015 with historical technologies embedded. We then examine the impact of removing improved crop varieties deployed in developing countries, thereby isolating their impact on the pattern of fine-scale LUC. Our findings show considerable complexity in the impacts of improved crop technologies at different spatial scales. At the global level, we see a reduction in cropland use from these technology improvements leading to gains in terrestrial carbon stock and avoided loss of threatened plant and animal species. In those locations which benefited the most from these technologies, crop profitability increases which leads to greater returns to land and further cropland expansion, thereby providing support for Jevon’s paradox. However, we also find that in other regions market-mediated spillover effects lower prices and returns to cropland, relative to the baseline, which leads to slower cropland expansion. Overall, we see less cropland expansion in areas of the world which are rich in biodiversity and terrestrial carbon which suggests that improved crop technologies contributed to overall gains in these environmental metrics. Given these findings, continued investments in agricultural research, including by the private sector as well as by national and international research centers, can help sustain agricultural productivity growth across the world, strengthen global food security, and mitigate agriculture’s environmental footprint in the coming decades.

## Materials and Methods

### SIMPLE-G Model.

The results in this study were generated using the SIMPLE-G modeling framework (Simplified International Model of agricultural Prices, Land use, and the Environment) a global-gridded partial equilibrium model of agriculture (48) (*SI Appendix*, *Supporting Text*). It focuses on the production and consumption of three aggregate food commodities (crops, livestock, and processed foods). In the model, consumption decisions are determined at the regional level and are driven by changes in population and per capita incomes. Regional consumer demands are also driven by economic responses to changes in incomes and commodity prices (i.e., income and price elasticities). Production decisions are modeled following the Constant Elasticity of Substitution (CES) production function (49) allowing cost-minimizing producers respond to changes in output and input prices. Under this framework, input substitution is determined by the changes in relative input prices and the elasticities of input substitution. Regional livestock and processed foods sectors use aggregate crop and noncrop inputs. In the particular version of SIMPLE-G which was developed for this work, regional crop supply is aggregated to crop production in about 120,000 grid cells with 0.25 × 0.25 degree resolution (~27 × 27 km near the equator). This allows SIMPLE-G to explicitly compute changes in crop output, cropland, and nonland input use at the fine scale. It also permits tracking of local changes in environmental outcomes—specifically terrestrial carbon stock as well as potential species loss.

In this study, cropland supply curves are defined at the grid cell level. Following the approach of Eickhout et al. (18), used in the MAGNET model (17), cropland supply in each grid is defined as a function of cropland rental rates and cropland supply asymptotes which is the maximum cropland in each grid cell. We use historical estimates of potentially cultivable lands (i.e., 1980–2009) for rainfed agriculture from Schneider (19) to calibrate these cropland supply asymptotes. These include lands which are suitable for agriculture and for crop cultivation.

Consumption of food commodities is estimated at the regional level and is driven by exogenous growth in population, income, and feedstock demand from biofuel mandates. Consumption is also a function of endogenous price changes. Changes in dietary patterns are captured by linking price and income elasticities of demand to the evolution of per capita income in each region. Prices are determined by the market clearing condition whereby supply is equal to demand at the regional level, with global exports also equaling global imports of crops. Market clearing for livestock and processed foods occurs at the regional level. For crops, consumers and producers tap both domestic and global markets as they respond to the relative prices of locally produced crops vs. crops purchased or sold in the world market. This follows the Armington trade model (50, 51) by allowing imperfect substitution between products in the local and global markets but without fully incorporating bilateral trade flows across regions.

The database constructed for this study is for circa 2015 (*SI Appendix*, *Supporting Text*). We heavily rely on the Global Trade Analysis Project (GTAP) v.10 database (Y2014) in calculating key economic shares (52) for each 17 region in SIMPLE-G. These include input cost shares for livestock and processed food commodities, crop utilization shares, and producer revenue as well as consumer expenditure shares for crops in the domestic and global markets. Input cost shares for crop production are calculated at the 160 GTAP regions and downscaled in each grid cell. The regional crop allocation shares for the biofuel sector are taken from GTAP-BIO database (53). The database also uses producer prices, crop output, and harvested area from FAOSTAT (1). Output data on 161 crops are aggregated using the ratio of producer prices to the global price of corn which is the numeraire in the model (i.e., metric tons of crop production are measured in corn-equivalent terms). To establish the initial pattern of production, regional crop output data are downscaled using spatial data on cropland extent as well as crop production maps. Subsequent changes in output are determined by the gridded economic model. Cropland extent data for 2015 at the gridded level are based on from Land Use Harmonization Database (v.2) (22). The distribution of crop production is constructed using grid cell level data from Monfreda et al. (21) which is calibrated for circa Y2000, national-level data from FAOSTAT (1) for Y2015 and distribution of cropland extent data for Y2015.

### Environmental Modules.

Fine-scale changes in cropland use from SIMPLE-G model are linked to spatial data on terrestrial carbon stock and land characterization factors. We use carbon stocks calculated by West et al. (54) to quantify the GHG emissions released into the atmosphere when cropland expands into natural lands. These estimates are based on spatially explicit datasets on potential vegetation and soil carbon. Note that these are one-time carbon emissions and do not account for the foregone carbon sequestered if the natural land remains untouched. Changes in biodiversity outcomes are computed using land occupation characterization factors from Chaudhary et al. (20) which represent potential biodiversity losses from using land in agriculture. These factors are calculated using the countryside species−area relationship model and data on species vulnerability. Five factors are calculated for mammals, birds, amphibians, and reptiles as well as plants species. Note that these factors are explicitly defined for 804 terrestrial ecoregions and for different land use conversion types. These factors also consider the degree of extinction risk faced by species within each ecoregion. For this study, we use land occupation characterization factors based on intensive use of land in crop production.

### Experimental Design.

The experimental design involves simulating a historical baseline and a counterfactual scenario without GR productivity impacts for the periods 1961–1985, 1985–2000, and 2000–2015 (*SI Appendix*, Fig. S1). Historical growth rates in regional population (1), per capita incomes (1), biofuels, total factor productivity (TFP) for crops and livestock (55), and TFP for the processed food sector (56) are used (*SI Appendix*, Table S1). Grid level changes in cropland cover (22) are also used to shift land supply in each grid cell in both baseline and counterfactual scenarios. For the counterfactual scenario, we subtract the grid-level TFP gains attributed to adoption of improved varieties (aggregated across three major food crops) (10) from the regional historical (baseline) TFP growth rate of the crop sector (55). Specifically, we downscale these TFP estimates using gridded crop production maps from Monfreda et al. (21), national-level data from FAOSTAT (1) for Y2015 and distribution of cropland extent data for Y2015 (*SI Appendix*, *Supporting Text*). To obtain the 95% CIs on our results, we use the R package “EnvStats” (57) to implement Latin Hypercube sampling (n = 375) for selected economic response parameters, terrestrial carbon emission factors, and biodiversity land characterization factors (*SI Appendix*, Table S2).

## Supplementary Material

Appendix 01 (PDF)

## Data Availability

The full model code and inputs supporting the manuscript’s results are available in Zenodo (https://doi.org/10.5281/zenodo.13631760) (58).

## Footnotes

^*^The CGIAR (formerly the *Consultative Group for International Agricultural Research*, now just known by its acronym) was constituted in 1971 by a group of donors to coordinate financial support for an emerging collection of international agricultural research centers, the first of which was established in 1960. Today the CGIAR system consists of 13 centers engaged in agricultural research with a focus on developing countries and recently reorganized as *One CGIAR*.
