# Supplementary material for: Adoption of improved crop varieties limited biodiversity losses, terrestrial carbon emissions, and cropland expansion in the tropics
Source: Proc Natl Acad Sci U S A. 2025 Feb 3;122(6):e2404839122. doi: 10.1073/pnas.2404839122 (PMC11831202; doi:10.1073/pnas.2404839122)
Supplement: Supplementary file 1 — Appendix 01 (PDF) [file pnas.2404839122.sapp.pdf]

PNAS

Supporting Information for

Adoption of improved crop varieties limited biodiversity losses, terrestrial carbon emissions and cropland expansion in the tropics

Uris Lantz C. Baldos[a,1]
Alfredo Cisneros Pineda[a]
Keith O. Fugle[b]
Thomas W. Hertel[a]

Authors Affiliation: [a]Department of Agricultural Economics, Purdue University, West Lafayette, IN 47907; [b]USDA Economic Research Service, Washington, DC 20250
[1]To whom correspondence may be addressed Email: ubaldos@purdue,edu

This PDF file includes:

Supporting text
I. Documentation of SIMPLE-G model and database 2-3
II. Mathematical Description of SIMPLE-G Model 4-7
III. Regional vs. Gridded Outcomes 8
IV. Contribution of TFP shocks by commodity 9
V. Commodity Aggregation and Co-Movements Among Commodity Prices 10-11
VI. The Impact of Soybean Variety Improvement in South America 12-13
Figures
Fig. S1. Summary of Experimental Design....14
Fig. S2. Estimated change (in %) in average crop total factor productivity due to diffusion of historical TFP growth from modern crop varieties over periods 1961 to 1985, 1985 to 2000, 2000 to 2015 and 1961 to 2015 15
Fig. S3. Changes in global crop production and prices due to adoption of improved crop and CGIAR technologies for periods 1961 to 1985, 1985 to 2000, 2000 to 2015 and 1961 to 2015 16
Fig. S4. Land supply asymptotes for Year 2015 17
Fig. S5. Regional changes in cropland area due to adoption of IV crop technologies for periods 1961 to 1985, 1985 to 2000, 2000 to 2015 and 1961 to 2015 18
Fig. S6. Changes in potential species loss due to adoption of IV crop technologies for periods 1961 to 1985, 1985 to 2000, 2000 to 2015 and 1961 to 2015 19
Fig. S7. Biodiversity hotspots for plants species 20
Fig. S8. Changes in potential species loss for plants due to adoption of IV crop technologies for period 1965 to 2015 for each biodiversity hotspot 21
Fig. S9. Average changes in total factor productivity, cropland use, LUC GHG emissions and potential species loss due to adoption of CGIAR crop technologies for period 1961 to 2015.... 22
Fig. S10. Changes in regional and global cropland use, biodiversity and terrestrial carbon from IV technology adoption: Regional vs Gridded models 23
Fig. S11. Regional change in TFP over the period 1965 to 2015 by major crop groups 24
Fig. S12. Long-term co-movements among crop commodity prices 25
Fig. S13. Economic benefits from adoption of improved varieties of soybeans in Brazil 26
Fig. S14. Soybean area harvested and soybean area under GM soybean in Brazil, 1961-2020. 27
Fig. S15. Potential contributions of IV adoption to soybean yield growth in South America 28
Fig. S16. Global changes in cropland use change, LUC GHG emissions and potential species loss due to IV adoption including potential contribution of soybean yield growth in South America for period 1961 to 2015 ...29
Fig. S17. Average changes in total factor productivity, cropland use, LUC GHG emissions and potential species loss due to IV adoption including potential contribution of soybean yield growth in South America for period 1961 to 2015....30
Tables
Tab. S1. Key per annum growth rates used for the historical and counterfactual simulations 31
Tab. S2. Distribution of key model parameters used in the uncertainty analysis using Latin Hypercube Sampling (n=375) 32
Tab. S3. TFP shocks from adoption of improved varieties of food crops by 2016-2020 33
Tab. S4. Simple correlations between crop commodity annual prices 34
Tab. S5. Adoption of Improved Varieties and by 2016–2020.... 35
Tab. S6. Share of Total Crop Area in Improved Varieties by 2016–2020.... 36
SI References 37-41

Supporting Information Text

I. Documentation of SIMPLE-G model and database

Background: The SIMPLE-G model is a global-gridded partial equilibrium model of agriculture. It is based on the analytical framework by Hertel (1) which examined the drivers as well as the key economic responses which govern long run changes in cropland demand and supply. Versions of SIMPLE-G model has been developed to examine issues on the water quality co-benefits of US climate policies (2), policies targeting nutrient pollution in the US corn production (3), transportation polices on local agricultural production and land use in Brazil (4) as well as the compounding effects of pandemic and weather stress on the global food systems (5). The version of SIMPLE-G developed for this study builds on the non-gridded version by Fuglie et al (6) and extends it to model of crop production as well as cropland supply at the fine scale.

There are three broad types of equations in the model: consumer demands, food and agricultural supplies, and commodity market clearing. In the model, consumption decisions are determined at the regional level and are driven by changes in population and per capita incomes. Per capita consumer demands are simple log-linear relationships in which the own-price and income elasticities vary as a function of per capita income level. Production decisions are modeled following the Constant Elasticity of Substitution (CES) production function (7) allowing cost-minimizing producers respond to changes in output and input prices. Under this framework, input substitution is determined by the changes in relative input prices and the elasticities of input substitution. There are several market clearing conditions in this model. For crops, market clearing occurs at the regional level and at the global scale. For livestock and processed foods, market clearing happens in each region. Some equations in the model are readily expressed in linearized form (i.e. percentage change, see II. Mathematical Description of SIMPLE-G). Note that when this model is solved with the linearized-levels variable linkages, we obtain the same solution as would be obtained by implementing the model in levels form. For more details on this mixed, linearized-levels representation of an economic model, see Hertel et al (8).

Base Data for Y2015: Creating the data for year 2015 requires several steps. First, we start by calculating total value of crop production and total crop output for each country using price and quantity data from FAOSTAT (9). In SIMPLE-G, total crop output is expressed in "corn-equivalent" which is calculated by deflating each crop output by the ratio of its world crop price to the world price of corn. To share-out crop value, output and harvested data from the country-level to the grid-level, we start with the gridded crop production and harvested area data from Monfreda et al (10) for circa Y2000. For each crop, we then calculate the subnational crop output and harvested area using boundary maps from the Global Administrative Maps (11). We then update the subnational crop output and harvested area for each crop by sharing out country-level data from FAOSTAT (9) for Y2015. We also calculate subnational cropland area using data from Land Use Harmonization Database (v.2)(12) for Y2015. We then use the ratio between harvested area to cropland area (i.e. cropping intensity) at the subnational level as well as gridded cropland area to downscale the subnational harvested area for each crop to each grid cell. This process ensures that the harvested area data and production data follows the spatial pattern of the cropland data. Using the gridded harvested area data as well as the subnational crop yield

data, we then downscale crop production for each crop at the grid level. We then calculate corn equivalent crop production in each grid using the ratio of world crop price to the world price of corn. To ensure that gridded-level production and value adds up to the regional data in SIMPLE-G and to make it consistent with the rest of the database, we downscale the region-level crop production and value data using crop production and value data in each grid cell as weights. With value of crop production at the grid level, we then use country-level cost share information for the crop sector from GTAP V.10 (13) for year Y2014 as well as the zero profit condition in each grid cell to initialize the value and quantity of non-land input use as well as value of land inputs in crop production.

The rest of the database is constructed as follows. On the demand side, the benchmark data include the value and quantity of crops used in direct food consumption, for feed in the livestock sectors, as raw inputs in the processed food industries, and as feedstocks in the biofuel sector. The amount of crop feedstock used by the biofuel sector in each region is calculated using the sales shares of the crop sector in the bioenergy sectors from the GTAPBIO V.9 database (14). Remaining crop quantities are allocated to food, feed, and processed food input use using crop purchase shares in the local and global markets using share data from the GTAP V.10 (13). The value of consumption for livestock and processed food commodities is equal to the total value of output for these sectors, given the assumption that total revenue is equal to total consumption expenditure for each commodity. Under zero-profit conditions, the total value of output is equal to the total production costs. The total cost of crop inputs in the livestock and processed food sectors is calculated using the crop prices and crop use quantities. The values of non crop inputs are then computed from the total cost of crop inputs and input cost shares. The input cost shares for livestock and processed food commodities are taken from the GTAP V.10 (13).

Fine-scale changes in cropland use from SIMPLE-G model are linked to spatial data on terrestrial carbon stock and land characterization factors. We use carbon stocks calculated by West et al. (15) to quantify the GHG emissions released into the atmosphere when cropland expands into natural lands. These estimates are based on spatially explicit datasets on potential vegetation and soil carbon. Note that these are one-time carbon emissions and do not account for the foregone carbon sequestered if the natural land remains untouched. Changes in biodiversity outcomes are computed using land occupation characterization factors from Chaudhary et al (16) which represent potential biodiversity losses from using land in agriculture. These factors are calculated using the countryside species−area relationship (CSAR) model and data on species vulnerability. Five factors are calculated for mammals, birds, amphibians, and reptiles as well as plants species. Note that these factors are explicitly defined for 804 terrestrial ecoregions and for different land use conversion types. These factors also consider the degree of extinction risk faced by species within each ecoregion. For this study, we use land occupation characterization factors based on intensive use of land in crop production.

Cropland supply curves are defined at the grid cell level. Following the approach of Eickhout et al (17), used in the MAGNET model (18), cropland supply in each grid is defined as a function of cropland rental rates and cropland supply asymptotes which is the maximum cropland in each grid cell. We use historical estimates of potentially cultivable lands (i.e. 1980-2009) for rainfed agriculture from Schneider (19) to calibrate these cropland supply asymptotes. These include lands which are suitable for agriculture and for crop cultivation.

II. Mathematical Description of SIMPLE-G Model

SETS

Commodities	i	Crops, Livestock, Processed Food, Non-Food
Food Commodities	$j \in J$<br>J={Crops,Lvstck,PFood}	Crops, Livestock, Processed Food
Crop Markets	$m \in M$<br>M={Local,Global}	Local, Global
Regions	r	Eastern Europe, North Africa, Sub Saharan Africa, South America, Brazil, Australia + New Zealand, European Union, South Asia, Central America + Caribbean, South Africa, South East Asia, Canada, United States, China, Middle East, Japan + Korea, Central Asia
Grids	g	n = 120306, 0.25 x 0.25 degree grids mapped to each region r

PARAMETERS

Price elasticity of commodity demand	$\varepsilon_{P(i,r)}$	Calculated using estimates from Muhammed et al (20)
Income elasticity of commodity demand	$\varepsilon_{Y(i,r)}$	Calculated using estimates from Muhammed et al (20)
Input substitution elasticity	$\sigma_{(\text{Crops},g)}$<br>$\sigma_{(\text{Lvstck},r)}$<br>$\sigma_{(\text{PFood},r)}$	Crops: Calibrated using US output price yield response from Keeney and Hertel (21). Uniform value<br>Livestock: Calibrated from Historical Simulations in Baldos and Hertel (22) . Uniform value<br>Processed Foods: Leontief Production Function
Cropland supply response to cropland rents	$v_{L(g)}$	Calculated using approach of Eickhout et al (17), used in the MAGNET model (18), existing cropland area from Land Use Harmonization Database (v.2) (12) and historical estimates of potentially cultivable lands (i.e. 1980-2009) for rainfed agriculture from Schneider et al (19)
Land elasticity parameter	$W_{L(g)}$	Starting value at 0.5 and calibrated for some grid cells to ensure cropland supply elasticities are between [0.01 to 5.00]
Non-land supply response to non-land prices	$v_{NL(g)}$	Calculated from Keeney and Hertel (23) and scaled up using long run cropland supply response from Ahmed, Hertel & Lubowski (24). Uniform value
Demand substitution elasticity for crops in the global and local markets	$\sigma_{SUBD(r)}$	Calibrated from Armington substitution parameters from GTAP V.10 (13). Uniform value
Supply transformation elasticity for crops in the global and local markets	$\sigma_{TRAN(r)}$	Calibrated from Armington substitution parameters from GTAP V.10 (13). Uniform value
Terrestrial carbon stocks in potential vegetation	$C_{(g)}$	Gridded data from West et al (15)
Land Occupation Factors	$O_{(g)}$	Data from Chaudhary et al (15) by ecosystem region and mapped to each grid in SIMPLE-G, explicit for mammals, birds, amphibians, and reptiles as well as plants species

SHARES

Cost share of croplands	$\theta_{L(g)}$	Country-level data from GTAP V.10 (13) which is applied to all grid cells in each country
Cost share of non-land inputs	$\theta_{NL(g)}$	Country-level data from GTAP V.10 (13) which is applied to all grid cells in each country
Cost share of crop inputs in the processed food sectors	$\theta_{P(r)}$	Based on GTAP V.10 (13)

Cost share of non-crop inputs in the processed food sectors	$\theta_{NP(r)}$	Based on GTAP V.10 (13)
Cost share of crops used as feed in the livestock sectors	$\theta_{F(r)}$	Based on GTAP V.10 (13)
Cost share of non-crop inputs in the livestock sectors	$\theta_{NF(r)}$	Based on GTAP V.10 (13)
Share of crops purchased by consumers in the global and local markets	$\theta_{QDCROP(m,r)}$	Based on GTAP V.10 (13)
Share of crops supplied by producers in the global and local markets	$\theta_{QSCROP(m,r)}$	Based on GTAP V.10 (13)

VARIABLES

*Lower case letters indicate percentage change variables while upper case letters indicate level variables.

Per capita commodity demand	$Q_{PC(i,r)}$<br>$q_{PC(i,r)}$	Crops: Quantities calculated from FAOSTAT (9)<br>Livestock, Processed Foods and Non-Food: Value of Consumption taken from GTAP V.10 (13)
Regional commodity demand	$Q_{(i,r)}$<br>$q_{(i,r)}$	Computed from per capital demand and population variables
Population	$POP_{(r)}$<br>$pop_{(r)}$	FAOSTAT (9)
Per capita income	$Y_{PC(r)}$<br>$y_{PC(r)}$	FAOSTAT (9)
Cropland area	$X_{L(g)}$<br>$x_{L(g)}$	Land Use Harmonization Database (v.2) (12)
Maximum cropland area	$X_{L(g)}^{MAX}$<br>$x_{L(g)}^{MAX}$	Historical estimates of potentially cultivable lands (i.e. 1980-2009) for rainfed agriculture from Schneider et al (19)
Non-land input quantity	$X_{NL(g)}$<br>$x_{NL(g)}$	Computed from zero profit condition in crop sector
Feeds used in the livestock sectors	$X_{F(r)}$<br>$x_{F(r)}$	Computed FAOSTAT (9) and crop utilization shares from GTAP V.10 (13)
Non-crop inputs used in the livestock sectors	$X_{NF(r)}$<br>$x_{NF(r)}$	Computed from zero profit condition in livestock sector
Crop inputs used in the processed food sectors	$X_{P(r)}$<br>$x_{P(r)}$	Computed FAOSTAT (9) and crop utilization shares from GTAP V.10 (13)
Non-crop inputs used in the processed food sectors	$X_{NP(r)}$<br>$x_{NP(r)}$	Computed from zero profit condition in processed food sector
Crop feedstock used in the global biofuel sector	$X_{BIO(r)}$<br>$x_{BIO(r)}$	Crop biofuel utilization shares from Taheripour et al (14)
Consumer demand for crops in the global and local markets	$QD_{CROP(m,j,r)}$<br>$qd_{CROP(m,j,r)}$	Computed FAOSTAT (9) and crop utilization shares from GTAP V.10 (13)
Producer supply for crops in the global and local markets	$QS_{CROP(m,r)}$<br>$qs_{CROP(m,r)}$	Computed FAOSTAT (9) and crop utilization shares from GTAP V.10 (13)
Producer supply for crops	$Q_{CROP(r)}, Q_{CROP(g)}$<br>$q_{CROP(r)}, q_{CROP(g)}$	Based on FAOSTAT (9)
Input neutral productivity change parameter	$ao_{(\text{Crops},g)} ao_{(\text{Lvstck},r)} ao_{(\text{PFood},r)}$	
Consumer price of commodity	$p_{D(i,r)}$	

Producer price of commodity	$p_{S(j,r)}$
Cropland rent	$r_{L(g)}$
Non-land prices	$r_{NL(g)}$
Price of non-crop inputs in the processed food sectors	$r_{NP(r)}$
Price of non-crop inputs in the livestock sectors	$r_{NF(r)}$
Price for crops in the local markets	$p_{CROPL(r)}$
Price for crops in the global markets	p_{CROPW}
Cropland Supply shifter	$s_{L(g)}$
Non-land Supply shifter	$s_{NL(g)}$
Change in terrestrial carbon stock	$CSTOCK_{(g)}$
Change in potential species loss	$PLOSS_{(g)}$

EQUATIONS

*Lower case letters indicate percentage change variables which upper case letters indicate levels variables

Per capita commodity demand	$q_{PC(i,r)} = \varepsilon_{P(i,r)} p_{D(i,r)} + \varepsilon_{Y(i,r)} y_{PC(r)}$
Regional commodity demand	$q_{(i,r)} = q_{PC(i,r)} + pop_{(r)}$
Derived demand for cropland inputs by crop sector	$x_{L(g)} = q_{CROP(g)} - ao_{(\text{Crops},g)} - \sigma_{(\text{Crops},g)}[r_{L(g)} - p_{S(\text{Crops},g)} - ao_{(\text{Crops},g)}]$
Derived demand for non-land inputs by crop sector	$x_{NL(g)} = q_{CROP(g)} - ao_{(\text{Crops},g)} - \sigma_{(\text{Crops},g)}[r_{NL(g)} - p_{S(\text{Crops},g)} - ao_{(\text{Crops},g)}]$
Zero profit condition in crop sector	$p_{S(\text{Crops},g)} + ao_{(\text{Crops},g)} = \theta_{L(g)} r_{L(g)} + \theta_{NL(g)} r_{NL(g)}$
Link between regional to gridded crop price changes for each grid ***g*** located in region ***r***	$p_{S(\text{Crops},g)} = p_{S(\text{Crops},r)}$
Link between regional to gridded crop quantities for each grid ***g*** located in region ***r***	$Q_{CROP(r)} = \sum Q_{CROP(g)}$
Cropland supply	$x_{L(g)} = v_{L(g)} r_{L(g)} - [X_{L(g)}^{MAX}/X_{L(g)}] * x_{L(g)}^{MAX} + s_{L(g)}$
Cropland supply elasticity	$v_{L(g)} = W_{L(g)} * [X_{L(g)}^{MAX}/X_{L(g)} - 1]$
Non-land supply	$x_{NL(g)} = v_{NL(g)} r_{NL(g)} + s_{NL(g)}$
Derived demand for crops in livestock sector	$x_{F(r)} = q_{(\text{Lvstck},r)} - ao_{(\text{Lvstck},r)} - \sigma_{(\text{Lvstck},r)}[p_{D(\text{Crops},r)} - p_{S(\text{Lvstck},r)} - ao_{(\text{Lvstck},r)}]$
Derived demand for non-crop inputs in livestock sector	$x_{NF(r)} = q_{(\text{Lvstck},r)} - ao_{(\text{Lvstck},r)} - \sigma_{(\text{Lvstck},r)}[r_{NF(r)} - p_{S(\text{Lvstck},r)} - ao_{(\text{Lvstck},r)}]$
Zero profit condition in livestock sector	$p_{S(\text{Lvstck},r)} + ao_{(\text{Lvstck},r)} = \theta_{F(r)} p_{D(\text{Crops},r)} + \theta_{NF(r)} r_{NF(r)}$
Derived demand for crops in processed food sector	$x_{P(r)} = q_{(\text{PFood},r)} - ao_{(\text{PFood},r)} - \sigma_{(\text{PFood},r)}[p_{D(\text{Crops},r)} - p_{S(\text{PFood},r)} - ao_{(\text{PFood},r)}]$
Derived demand for non-crop inputs in processed food sector	$x_{NP(r)} = q_{(\text{PFood},r)} - ao_{(\text{PFood},r)} - \sigma_{(\text{PFood},r)}[r_{NP(r)} - p_{S(\text{PFood},r)} - ao_{(\text{PFood},r)}]$
Zero profit condition in processed food sector	$p_{S(\text{PFood},r)} + ao_{(\text{PFood},r)} = \theta_{P(r)} p_{D(\text{PFood},r)} + \theta_{NP(r)} r_{NP(r)}$

Demand for crops in local market	$qd_{CROP(\text{Local,Crops},r)} = q_{(\text{Crops},r)} - \sigma_{SUBD(r)}[p_{CROPL(r)} - p_{D(\text{Crops},r)}]$
Demand for crops in global market	$qd_{CROP(\text{Global,Crops},r)} = q_{(\text{Crops},r)} - \sigma_{SUBD(r)}[p_{CROPW} - p_{D(\text{Crops},r)}]$
Demand for crops by livestock sector in local market	$qd_{CROP(\text{Local,Lvstck},r)} = x_{F(r)} - \sigma_{SUBD(r)}[p_{CROPL(r)} - p_{D(\text{Crops},r)}]$
Demand for crops by livestock sector in global market	$qd_{CROP(\text{Global,Lvstck},r)} = x_{F(r)} - \sigma_{SUBD(r)}[p_{CROPW} - p_{D(\text{Crops},r)}]$
Demand for crops by processed food sector in local market	$qd_{CROP(\text{Local,PFood},r)} = x_{P(r)} - \sigma_{SUBD(r)}[p_{CROPL(r)} - p_{D(\text{Crops},r)}]$
Demand for crops by processed food sector in global market	$qd_{CROP(\text{Global,PFood},r)} = x_{P(r)} - \sigma_{SUBD(r)}[p_{CROPW} - p_{D(\text{Crops},r)}]$
Supply for crops in the local market	$qs_{CROP(\text{Local},r)} = q_{CROP(r)} + \sigma_{TRAN(r)}[p_{CROPL(r)} - p_{S(\text{Crops},r)}]$
Supply for crops in the global market	$qs_{CROP(\text{Global},r)} = q_{CROP(r)} + \sigma_{TRAN(r)}[p_{CROPW} - p_{S(\text{Crops},r)}]$
Market clearing for crops in local market	$QS_{CROP(\text{Local},r)} = \sum_{j\in J} QD_{CROP(\text{Local},j,r)}$
Market clearing for crops in global market	$\sum_{r\in R} QS_{CROP(\text{Global},r)} = \sum_{r\in R}\sum_{j\in J}\left(QD_{CROP(\text{Global},j,r)}\right) + +X_{BIO(r)}$
Consumer crop price	$p_{D(\text{Crops},r)} = p_{CROPL(r)}\theta_{QDCROP(\text{Local},r)} + \theta_{QDCROP(\text{Global},r)}p_{CROPW}$
Producer crop price	$p_{S(\text{Crops},r)} = p_{CROPL(r)}\theta_{QSCROP(\text{Local},r)} + \theta_{QSCROP(\text{Global},r)}p_{CROPW}$
Regional prices for livestock	$p_{S(\text{Lvstck},r)} = p_{D(\text{Lvstck},r)}$
Regional prices for processed foods	$p_{S(\text{PFood},r)} = p_{D(\text{PFood},r)}$
Fixed non-food consumer prices	$p_{D(\text{NFood},r)} = 0$
Fixed price of non-crop inputs in the processed food sectors	$r_{NP(r)} = 0$
Fixed price of non-crop inputs in the livestock sectors	$r_{NF(r)} = 0$
Change in terrestrial carbon stock	$CSTOCK_{(g)} = \Delta X_{L(g)}C_{(g)}$
Change in potential species loss	$PLOSS_{(g)} = \Delta X_{L(g)}O_{(g)}$

III. Regional vs. Gridded Outcomes

Past work which assess the implications of international agricultural research on global cropland use and terrestrial carbon have relied on highly aggregated models (25, 26). Regional-level analysis ignores the heterogeneity in the terrestrial carbon, biodiversity data as well as the productivity impacts of IV technology adoption due to the different composition of crops being grown in each grid cell. Figure S10 shows the results from the regional and the gridded models, specifically for cropland use, avoided biodiversity loss as well as avoided terrestrial carbon emissions. Here, the regional model is constructed by aggregating the gridded database used in this study and using cropland area in each grid cell to create regional average values for terrestrial carbon stocks, land occupation factors, which measures biodiversity loss, as well as productivity estimates from IV technologies.

Comparing the model estimates, the regional and gridded model give us very different regional and global results. Starting with cropland use, we see that the regional model predicts greater global savings in cropland use than under the gridded model, as a consequence of IV technology adoption. Land use savings are more likely given the uniform impact of IV technology adoption within a region. In contrast, due to the differential impact of IV technology as well as competition across grid cells within a region, the gridded model shows smaller savings in cropland use due to adoption of IV technologies. There are also differences where additional cropland expansion is expected to occur given adoption of improved crop technologies. In the regional model, very strong cropland expansion is observed in Sub Saharan Africa. With large land use savings, the avoided global land use change emissions are also significant. Furthermore, despite a larger estimate of avoided global cropland use, the global estimates of the avoided species loss are slightly smaller under the regional model than in the gridded model because it assumes a single regional average value for this environmental metric. Given these, we believe that a regional model is likely to overstate land use savings and avoided GHG emissions and, at the same time, understate the biodiversity impacts given productivity growth due to IV technology adoption compared to a gridded model.

IV. Contribution of TFP shocks by commodity

Table S3 provides additional information on the commodities contributing to crop TFP growth in major developing-country regions. The first part of the table shows the average improvement in TFP for different commodities and commodity groups due to adoption of improved varieties in 19 food crops. The second part of the table gives the crop value shares for these commodities, indicating the relative importance of the commodity to the whole crop sector.

In Sub-Saharan Africa, RTB crops made up 34 percent of total crop production in 2016-2020, and the 11 percent increase in TFP for these crops made up a large part of the growth in TFP in the whole crop sector. In Asia, large TFP shocks in cereals, RTB and legumes had a relatively small effect on TFP growth in the whole crop sector because these crops combined made up only 29 percent of the value of crop production in this region (other crops, including vegetables, fruits, and perennial crops like oil palm, made up the other 71 percent). Overall in developing countries, the TFP shock to cereal grains had the largest impact on improving TFP in the crop sector, due to both the large increase in TFP in cereals (13.9 percent) and its large value share in total crop production (21 percent). Table IV.1 also lists TFP shocks in four specific crops – wheat, rice, maize, and cassava. TFP improvement in cassava was especially important in Sub-Saharan Africa, where it consists a major food staple accounts for 12 percent of the value of all crop production. Similarly, TFP shocks in rice are especially important for Asia as are TFP shocks in wheat for CWANA.

Figure S11 shows the regional change in TFP over the period 1961 to 2015 due to the adoption of improved varieties and breaks down the contribution of major crop groups (Cereals, Legumes, Root, Tubers & Bananas). Here, we aggregated the country level data from Fuglie and Echeverria (27) based on the geographic regional aggregation in the SIMPLE-G model. Note that majority of the productivity growth from adoption of IV technologies are mainly driven by cereals except in Sub-Saharan Africa where roots, tubers, and bananas have had a major share of the impact on aggregate crop TFP. These estimates only consider the impact of public varieties of soybean in Brazil and South America, which by 2015 had largely been replaced by private GM varieties (Figure S11A). If we assume, however, that observed gains in farmer's soybean yields in these regions were primarily due to genetic gains, then the impact of IV adoption on crop TFP would likely be much larger (Figure S11B). See the SI section on soybean yield in South America for further discussion.

V. Commodity Aggregation and Co-Movements Among Commodity Prices

In our model, we only consider the production and demand for a single aggregate crop commodity. Our rationale for this aggregation in our long run model is that relative crop prices have generally remained stable in the long run, due to substitution possibilities in consumption and production. This section includes an analysis on the co-movement of crop prices, using long-term annual commodity price comparisons for major grains, oilseeds, and other crops from the IMF and other sources. Relative prices of major grains and oilseeds have remained quite stable over the last six decades despite significant fluctuations in overall price levels.

For this study, “crop production” is the aggregation of the 162 crop commodities listed in the FAOSTAT database (9). Commodities are aggregated each year using constant average global farmgate prices from 2014-2016, where prices are measured in purchasing-power-parity (PPP) dollars per metric ton. The PPP prices are derived by FAO and used to create its index of gross agricultural and crop, and animal product outputs. It is a measure of the volume of production, where quantities vary year-to-year while prices remain fixed. Using global average prices also avoids distortions from country-specific policies, such as commodity taxes and subsidies.

Using fixed prices is a valid means for quantity aggregation so long as the relative prices among commodities remain roughly stable over time. Constant relative price would be expected for commodities that are close substitutes in either consumption or production. For commodities that are not close substitutes, relative prices may show greater variability especially in the short run. But so long as relative prices return to long run means, using fixed prices will not introduce aggregation biases. However, changes in consumer preferences (i.e., greater preferences for feed grains for animal product consumption versus food grains for direct consumption) could introduce secular changes in relative prices among commodities.

Here we examine long-run co-movements among crop commodities at major points of international trade (Figure S12). The IMF Primary Commodity Database (28) provide annual and monthly commodity price series for major grains, oilcrops, sugar, tropical beverage crops, fiber crops, and selected fruits (bananas and oranges) starting from 1957. One omission is root and tuber crops, which are not widely traded internationally. To address this limitation, we collected monthly U.S. wholesale prices for potato frozen French fries (available since 1967) and weekly export prices for Thai cassava starch (tapioca), which have been published since 1990 by the Thailand Tapioca Starch Association (29). We focus on the stability of relative prices of various commodities and commodity groups relative to cereal grains. Price indexes for commodity groups are constructed using weighted average growth rates of the individual price components of the group, where the weights are the value shares of global trade.

Table S4 shows simple correlations among annual crop commodity prices and Figure S12 plots prices indices for cereal grains and oilcrops as well as crop prices relative to cereal grains over 1957 to 2023 (except for roots and tubers – see footnote to table). For major grains and oilseeds, annual prices have been highly correlated, with correlation coefficients consistently above 0.80. For other commodities, most price correlations are above 0.6, although tend to be lower for perennial crops. From plots of relative price movements in Figure SI.3, it appears that changes in relative prices occur mainly in the short run when substitution possibilities are more limited. Over the long run, commodity prices relative to cereal grains appear to revert to long run means. Oilcrop prices may have shown a slight

tendency to increase relative to cereal grain prices, especially for peanuts in the 1980s. The general stability of relative crop prices, at least for major crops at their major trading hubs, supports using fixed price weights for aggregating crops into one crop commodity to simplify analysis of long-term trends in global agricultural production and resource use.

VI. The Impact of Soybean Variety Improvement in South America

One limitation of Fuglie and Echeverria (27) is that they only included crops and regions for which CGIAR centers had breeding programs. This limited the coverage of soybean to Sub-Saharan Africa. However, soybeans have been expanding rapidly in South America and India as well. The exclusion of soybeans in these regions is an important gap in using the Fuglie-Echeverria data for assessing the broader (CGIAR and non-CGIAR) impacts of food crop improvement on land use in developing countries. This is especially so for Brazil, where soybean expansion has been associated with deforestation (31). It is less of an issue for India, where total cropland area has not increased for several decades and thus soybean expansion occurred as a result of increased multiple cropping or through crop substitution. Moreover, Nuthalapati et al. (2024) (55) show that varietal turnover in soybeans in India has been very slow and consequently little change in average farm yield has occurred. However, in South America, soybean yield more than doubled over 1965-2015 and significant varietal change has occurred. Since the 1950s, adapting soybean varieties to tropical conditions was a major objective for public agricultural research in Brazil (32, 33), although in the 2000s public varieties were largely replaced by private-sector varieties that had been genetically modified (GM) for herbicide tolerance (ISAAA) (38-51).

To partly rectify the gap in soybean variety impact in South America, we added to the Fuglie-Echeverria dataset information on the impacts of public soybean varietal improvement in Brazil. For this we were able to draw upon two previous assessments of the economic impact of public soybean research in this country: Ayres (32), who examined soybean development over 1955-1983, and Pardey et al. (33) whose study covered the years from 1981 to 2003. Figure S13 shows the estimated impacts from Ayres (32) and Pardey et al. (33) when converted into the prices and currency units used by Fuglie and Echeverria (27) and the 5-year average values over 1965-2005 that were added to their estimates of food crop productivity shocks.

However, we do not have sufficient information to extend the estimates of soybean variety change in Brazil beyond the years shown in Figure S13. In the 2000s, privately developed genetically-modified (GM) soybean varieties became widely adopted and have dominated soybean variety-type in Brazil and other countries in South America ever since (Figure S14). While information on new variety registrations is published by the Brazilian government, we are unable to find sufficient data to allow us to suitably model the productivity impact of these varieties. Available studies note that adoption of herbicide-tolerant GM soybeans may have introduced significant factor biases in input use and production costs, in addition to effects on yield. Bustos et al. (34) claimed GM soybean was strongly biased toward saving labor and capital and had very little impact on yield, and thus encouraged cropland expansion into new areas. However, Zalles et al. (35) and Trigo and Cap (36) show that in Brazil and Argentina, the no-till cultivation system that was facilitated by herbicide-tolerant varieties enabled most soybean expansion to occur through conversion of pastures into cropland and through double cropping soybean with grain crops. Moreover, Bustos et al. (34) only considered the cost-savings impact of GM traits and ignored the likely influence of soybean genetic improvement on yield, which more than doubled over 1965-2015.

To establish an upper bound on the productivity impact of soybean IV adoption in South America, we re-estimated the model assuming that varietal improvement was responsible for the average growth in farm yields in major soybean producing countries over 1961-2015. For this scenario we assumed that for Brazil, Argentina, Paraguay, Uruguay and Bolivia, soybean breeding and variety turnover resulted in a 1% annual genetic gain in farm yields. Further, we assumed a rate of variety turnover of 7 years in Brazil and 10 years for the rest of South America. As shown in Figure S15, with these assumptions, then IV adoption would largely account for observed soybean yield changes in South America. Using these assumptions about the impact of soybean IV adoption results in much large TFP growth in the crop sector of these countries (see Figure S11B).

Re-estimating our model results in slightly larger estimates of global land savings from IV adoption (see Figure S16) at around 16.27 [95% CI, 12.56 – 21.75] million hectares worldwide over the period 1961 to 2015. With greater land savings, retained global terrestrial carbon stocks is estimated at around 5.57 [95% CI, 3.87 – 7.57] billion metric tons CO2 equivalent while around 1,273 [95% CI, 759 – 1,837] threatened plants and animals species avoided extinction. Figure S17 shows the global maps of total factor productivity growth, land use changes, changes in terrestrial carbon stocks as well as changes in potential species loss over the period 1961 to 2015. Looking at South America, we see greater productivity growth from soybean IV adoption for this region compared to the base scenario (Figure S2). Greater productivity results in expansion of cropland use and additional GHG emissions from land conversion in this region.

Due to incomplete information on GM soybean varietal change and its impact on productivity and input use, our main results focus on the impact of improved non-GM soybean varieties adopted in Brazil prior to 2003. However, soybean yield continued to rise significantly following adoption of GM varieties, which, as the results reported above show, may have had a considerable land-savings effect. A more complete assessment of the impact of soybean genetic change on land use would need to give careful attention to both potential land-saving and labor-saving biases in this technological change.

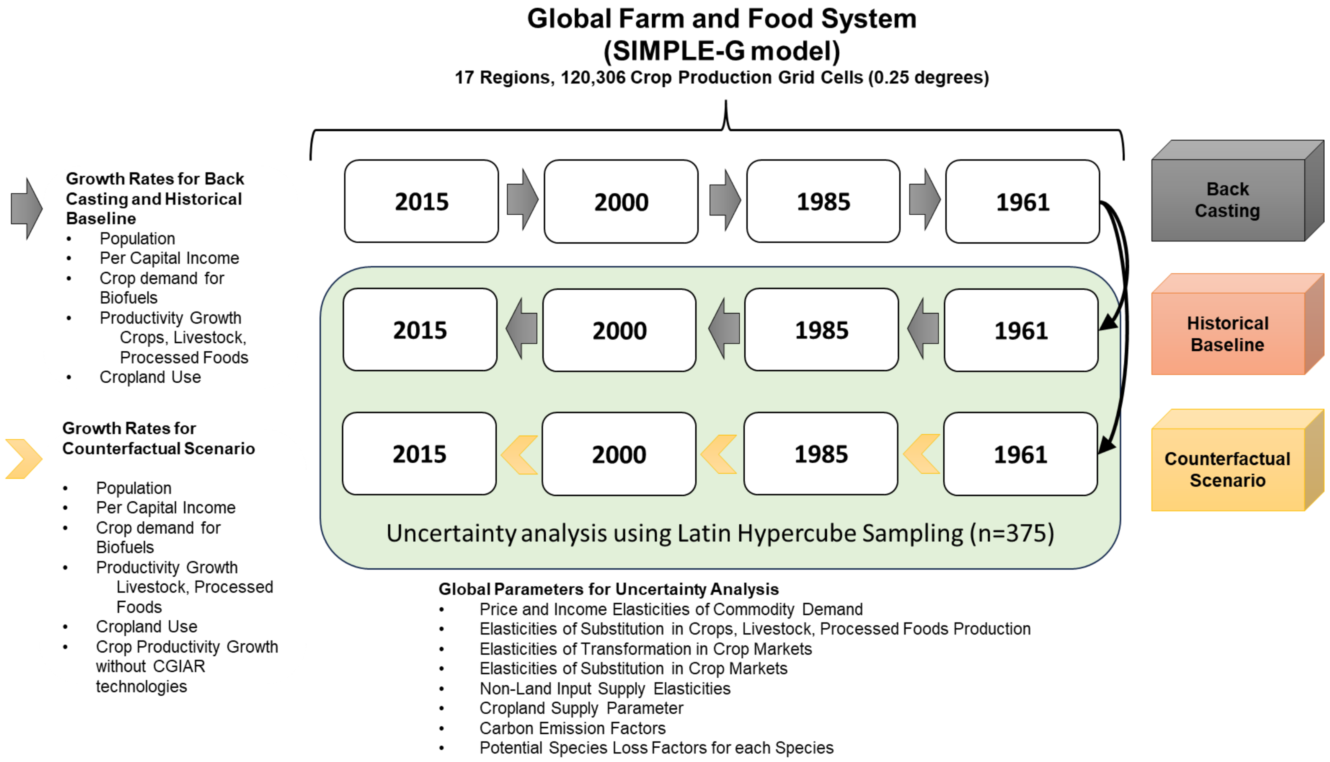

Fig. S1. Summary of Experimental Design. 'Back Casting' simulates the model back to year 1961 given historical growth rates of key model drivers. 'Historical Baseline' reproduce changes in world agriculture from 1961 to 2015 given 3 periods (1961-1985, 1985-2000, 2000-2015) while 'Counterfactual Scenario' projects world agriculture from 1961 to 2015 without the additional productivity growth from improved variety technologies. Uncertainty analysis is conducted using Latin Hypercube Sampling (n=375) for key global parameters which are scaled down to regional or grid-level values. For each sample, the Historical Baseline and the Counterfactual Scenario are simulated. Finally, the impacts of improved variety technologies are calculated by taking the difference in outcomes between the Historical Baseline and the Counterfactual Scenario.

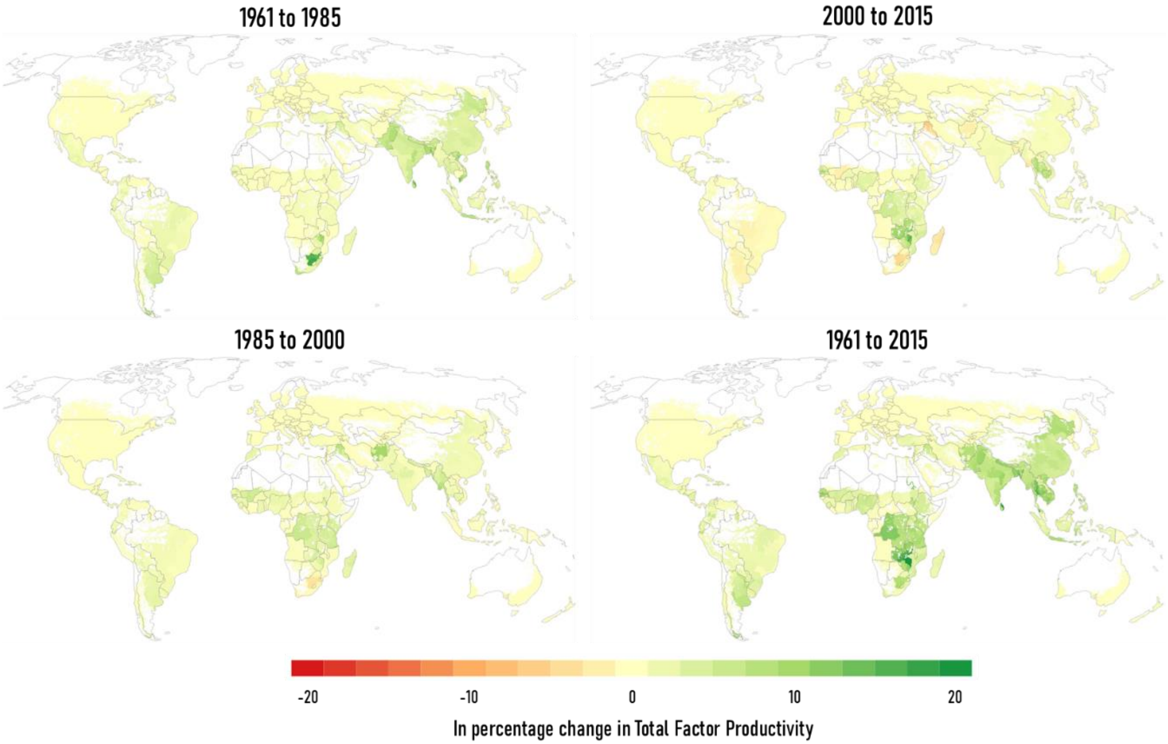

Fig. S2. Estimated change (in %) in average crop total factor productivity due to diffusion of historical TFP growth from modern crop varieties over periods 1961 to 1985, 1985 to 2000, 2000 to 2015 and 1961 to 2015. Darker shaded areas are where there was more intensive adoption of improved crop technologies and/or where productivity gains per hectare were high relative to crop yields without these new technologies. Red areas show reduction in productivity as existing IV varieties are replaced by those developed by national research programs or the private sector

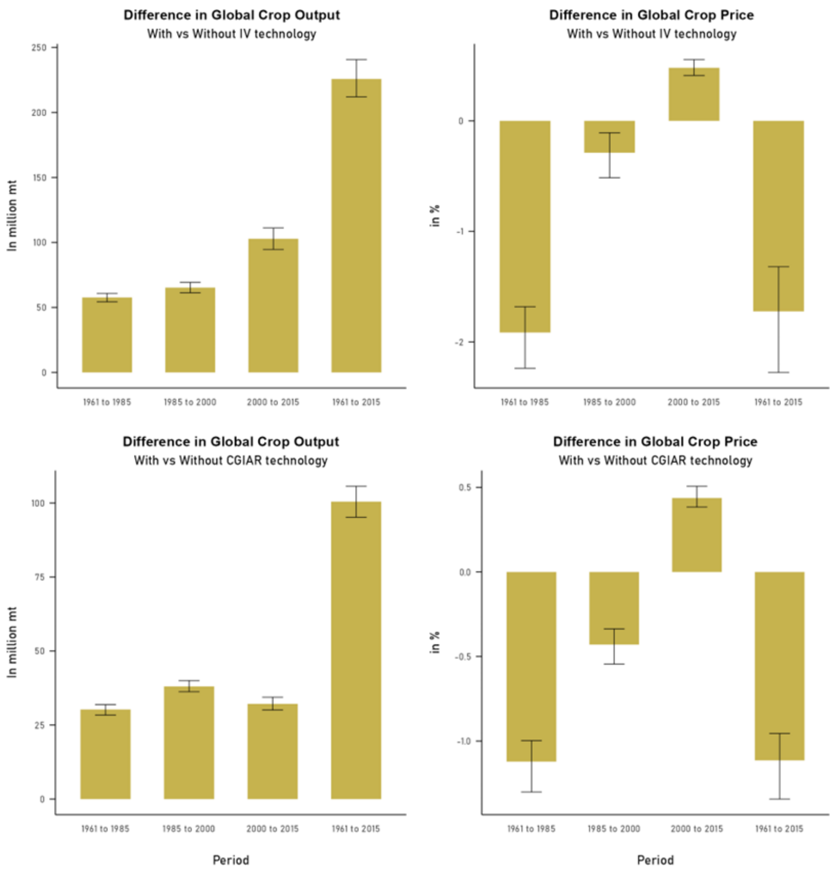

Fig. S3. Changes in global crop production and prices due to adoption of improved crop and CGIAR technologies for periods 1961 to 1985, 1985 to 2000, 2000 to 2015 and 1961 to 2015. Error bars show 2.5% and 97.5% percentiles.

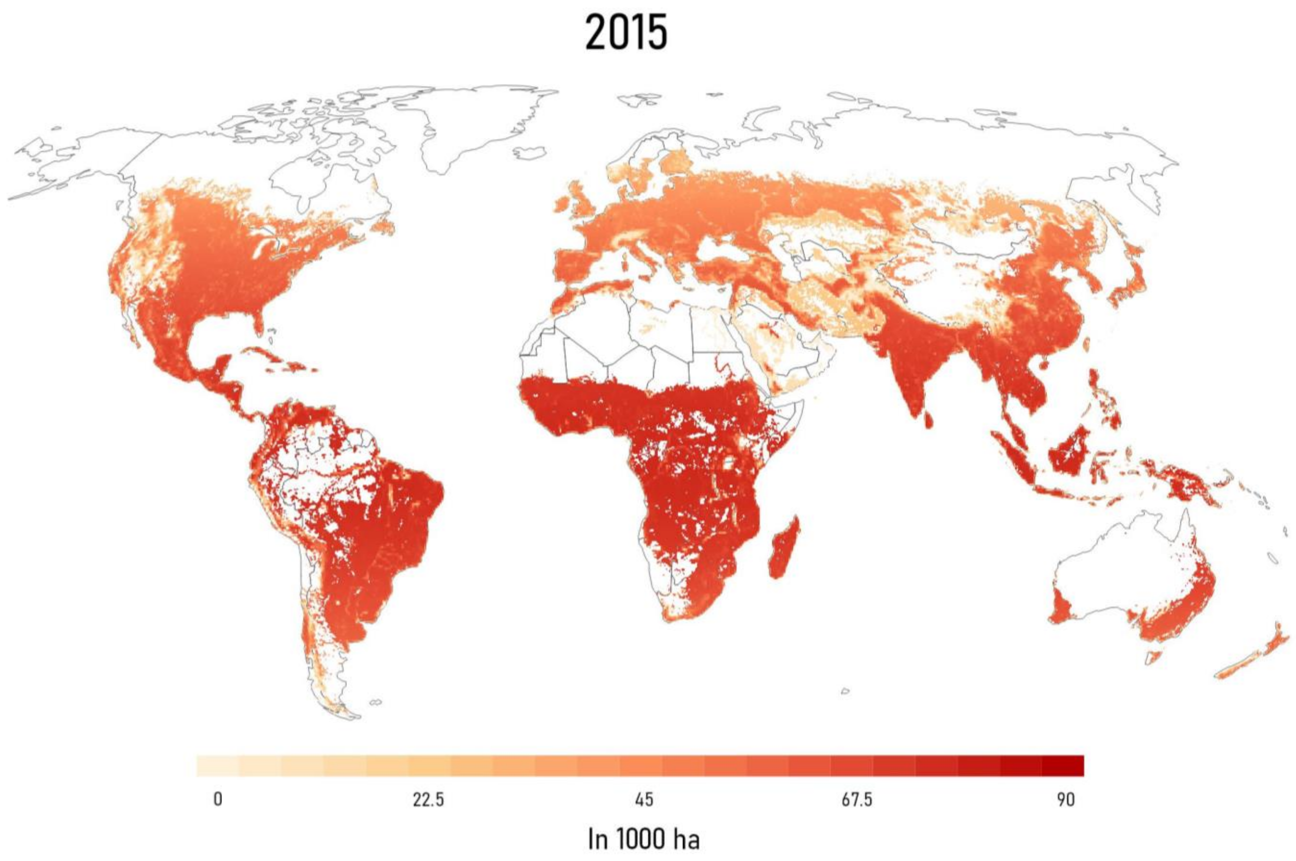

Fig. S4. Land supply asymptotes for Year 2015. These asymptotes define the maximum available cropland area in each grid cell and are constructed using cropland cover from Land-Use Harmonization (v.2) (12) **and historical estimates of potentially cultivable lands (i.e. 1980-2009) for rainfed agriculture from Schneider** (19)

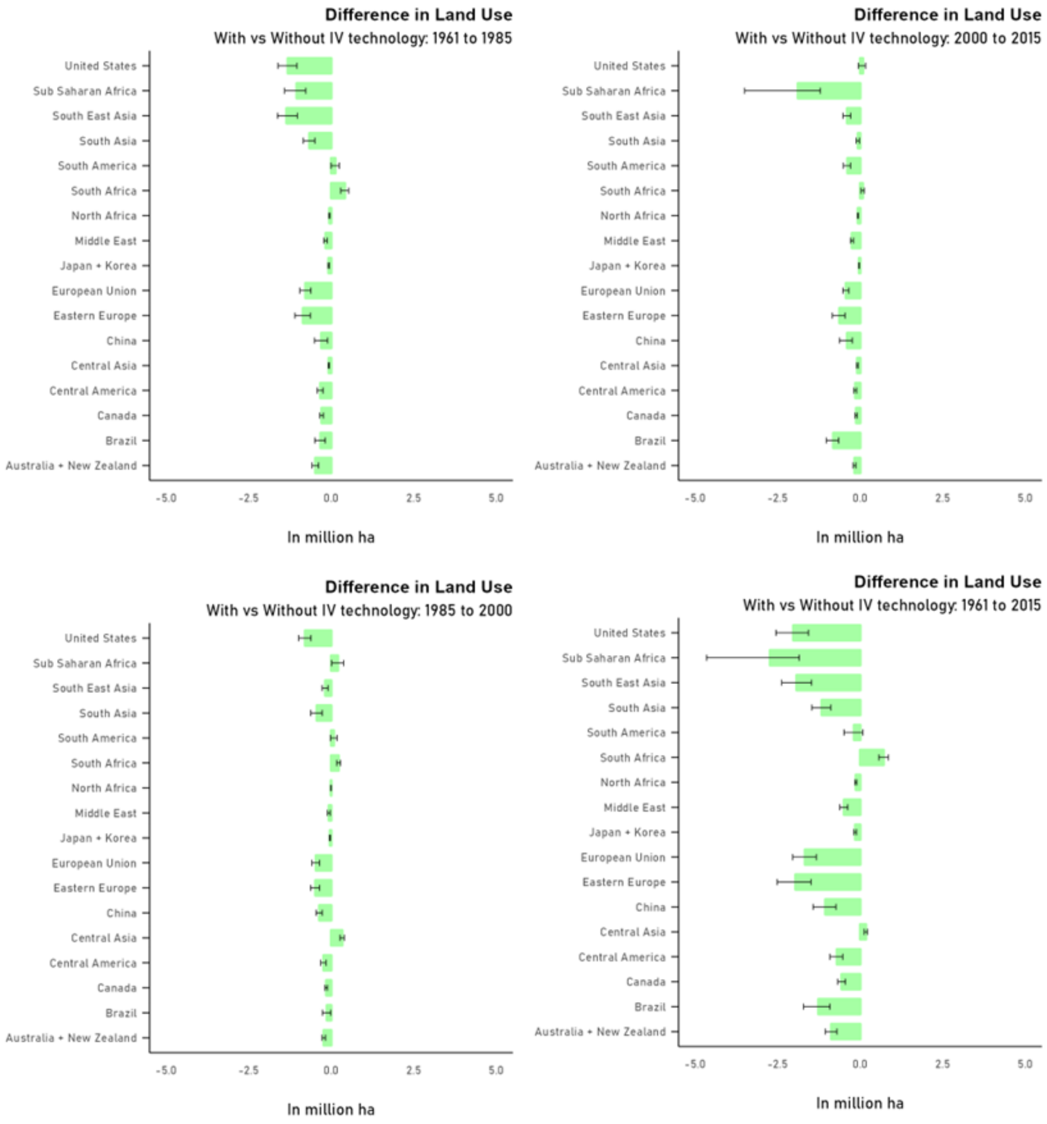

Fig. S5. Regional changes in cropland area due to adoption of IV crop technologies for periods 1961 to 1985, 1985 to 2000, 2000 to 2015 and 1961 to 2015. Error bars show 2.5% and 97.5% percentiles.

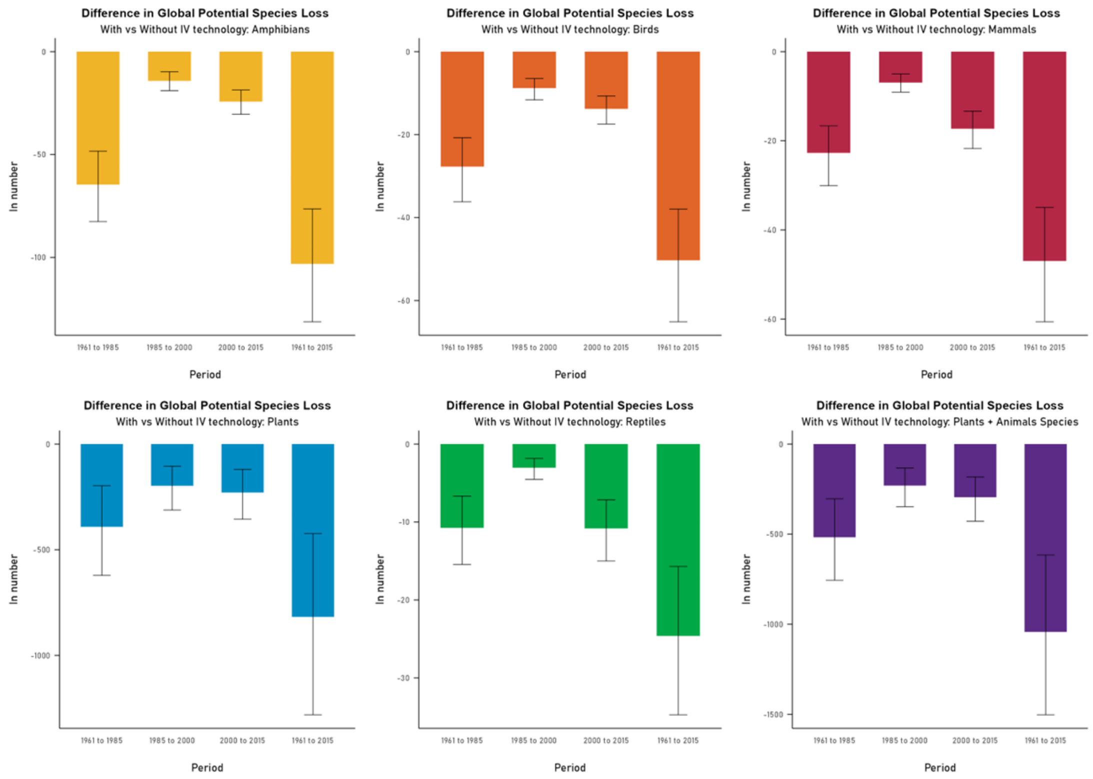

Fig. S6. Changes in potential species loss due to adoption of IV crop technologies for periods 1961 to 1985, 1985 to 2000, 2000 to 2015 and 1961 to 2015. Error bars show 2.5% and 97.5% percentiles.

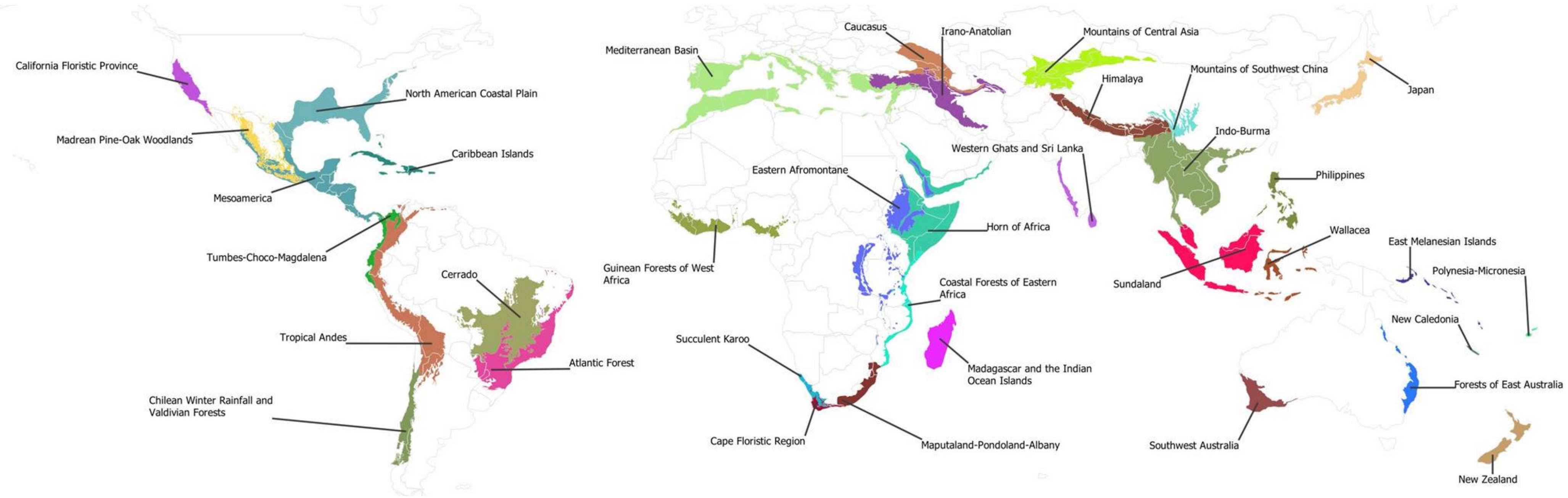

Fig. S7. Biodiversity hotspots for plants species. Hotspots are regions which has at least 1,500 plants as endemics (>0.5% of the world's total), and have 30% or less of its original vegetation remaining (52, 53)

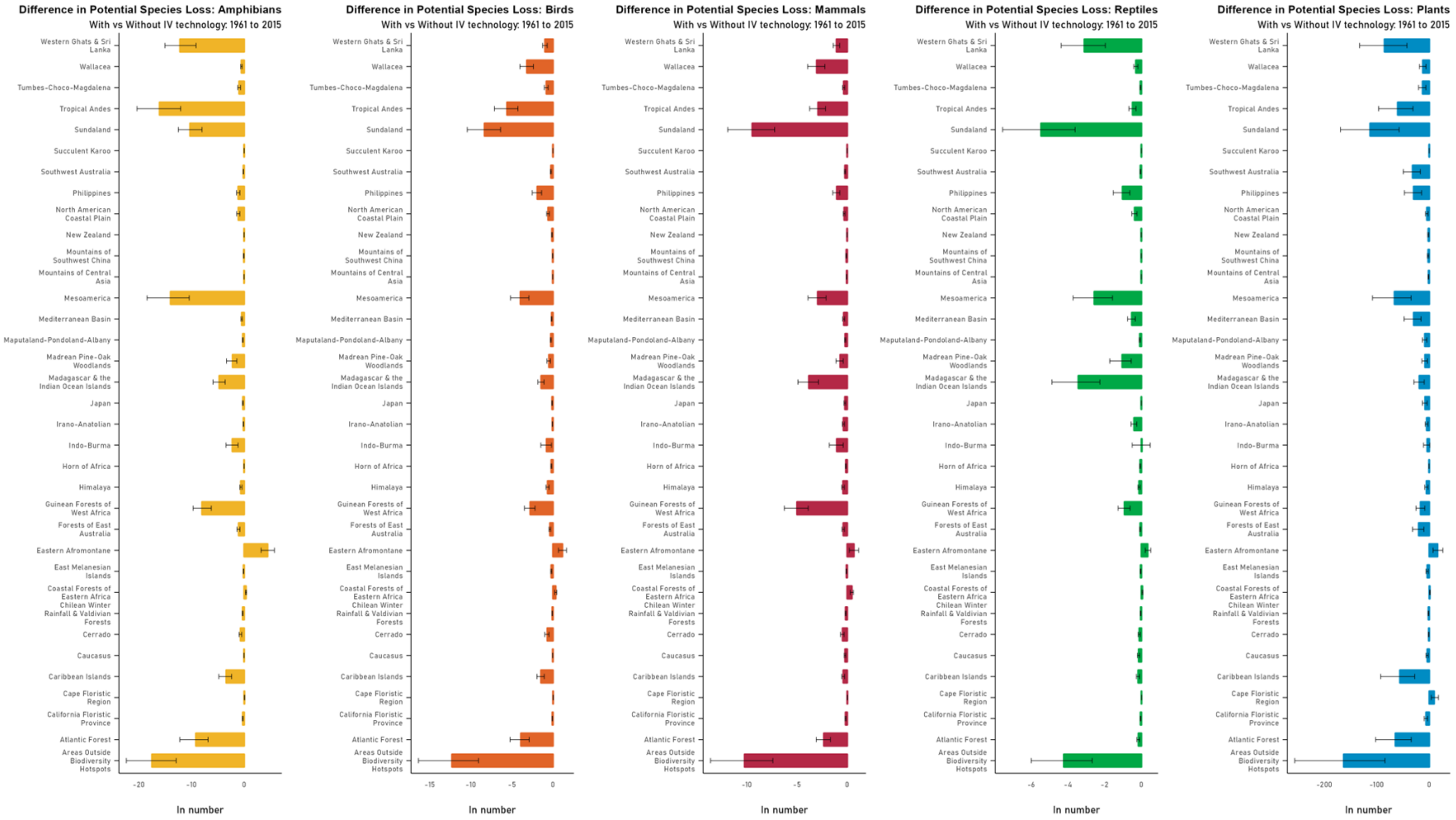

Fig. S8. Changes in potential species loss for plants due to adoption of IV crop technologies for period 1961 to 2015 for each biodiversity hotspot. Error bars show 2.5% and 97.5% percentiles.

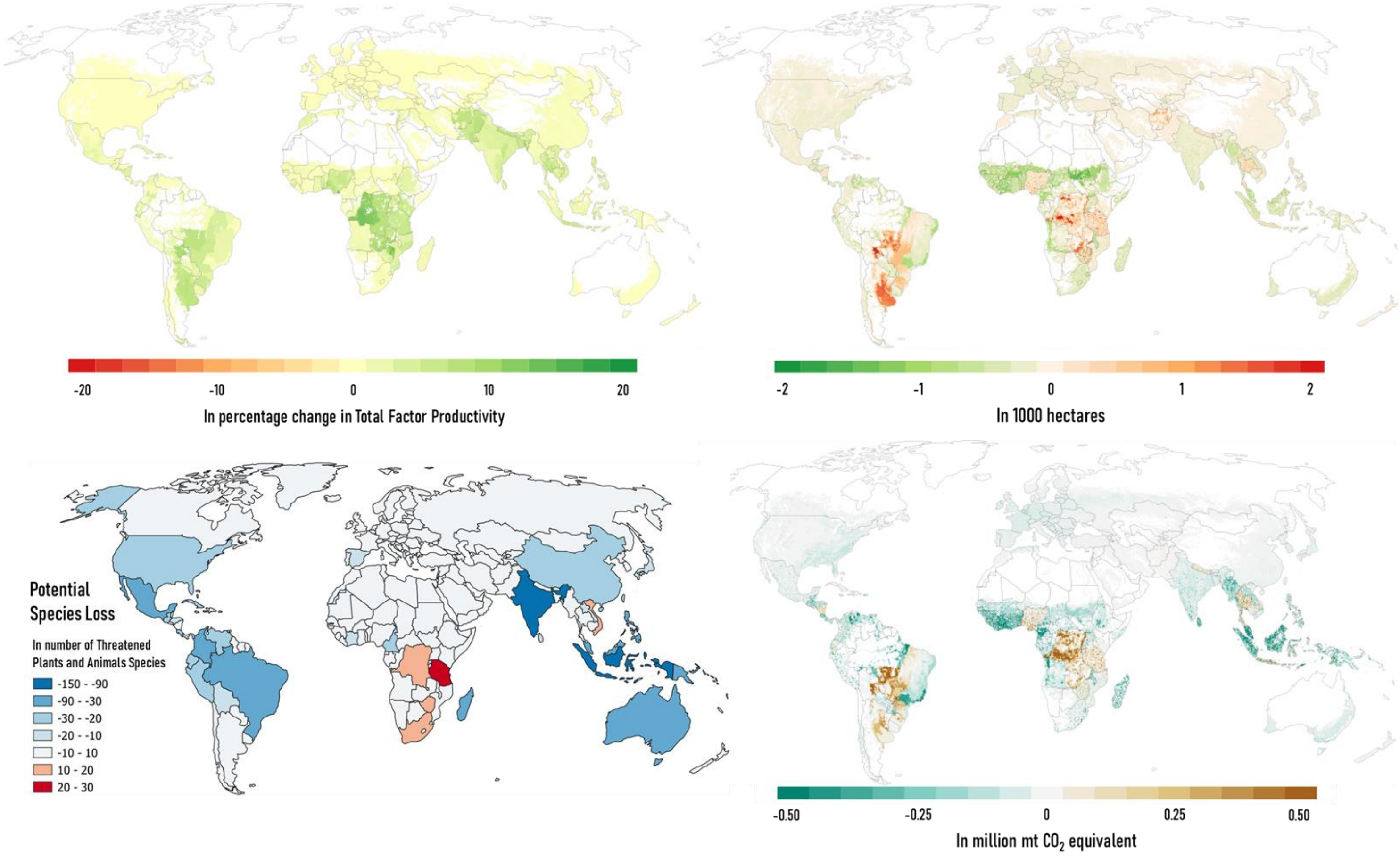

Fig. S9. Average changes in total factor productivity, cropland use, LUC GHG emissions and potential species loss due to adoption of CGIAR crop technologies only for period 1961 to 2015.

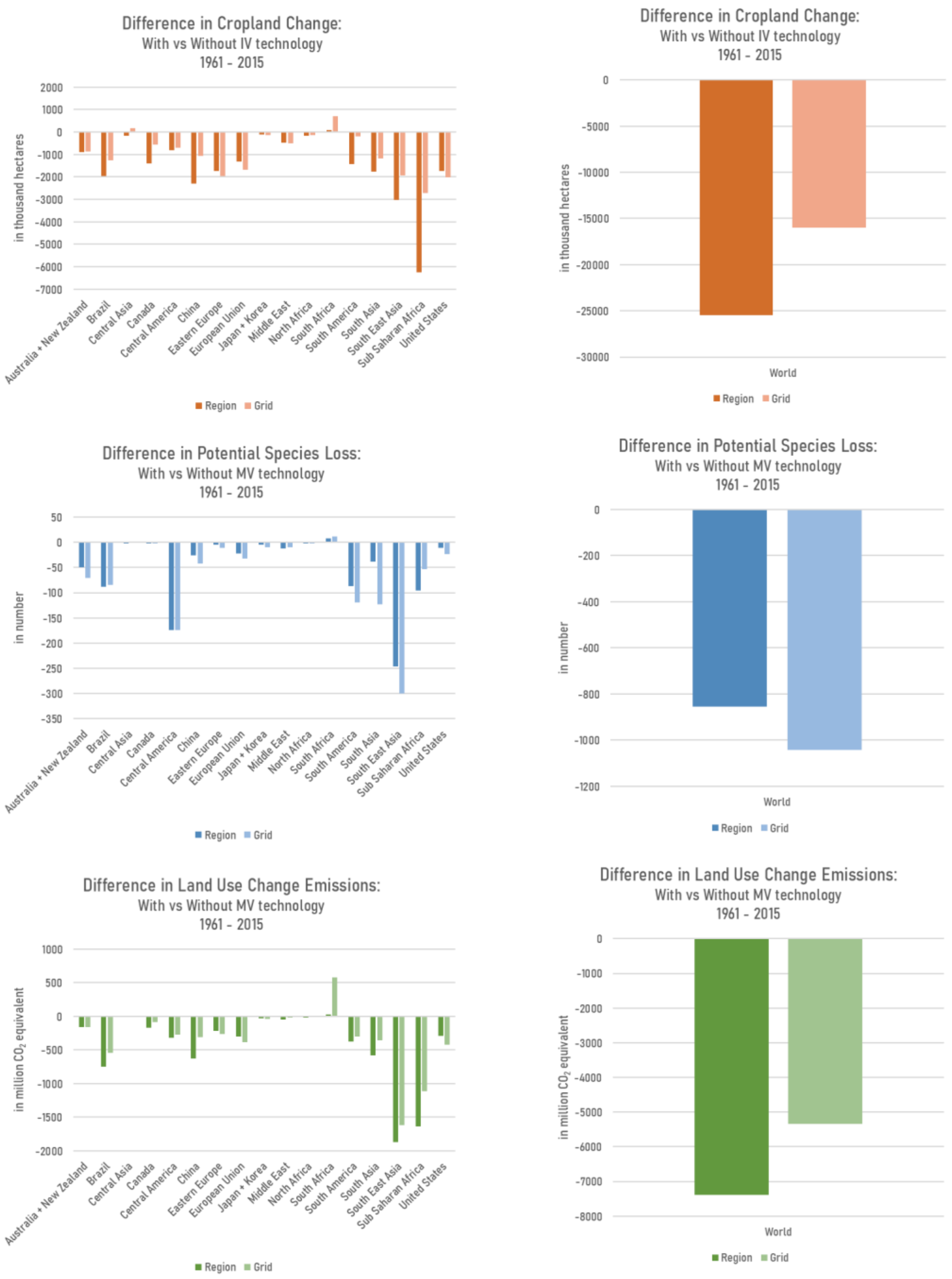

Fig. S10. Changes in regional and global cropland use, biodiversity and terrestrial carbon from IV technology adoption: Regional vs Gridded models.

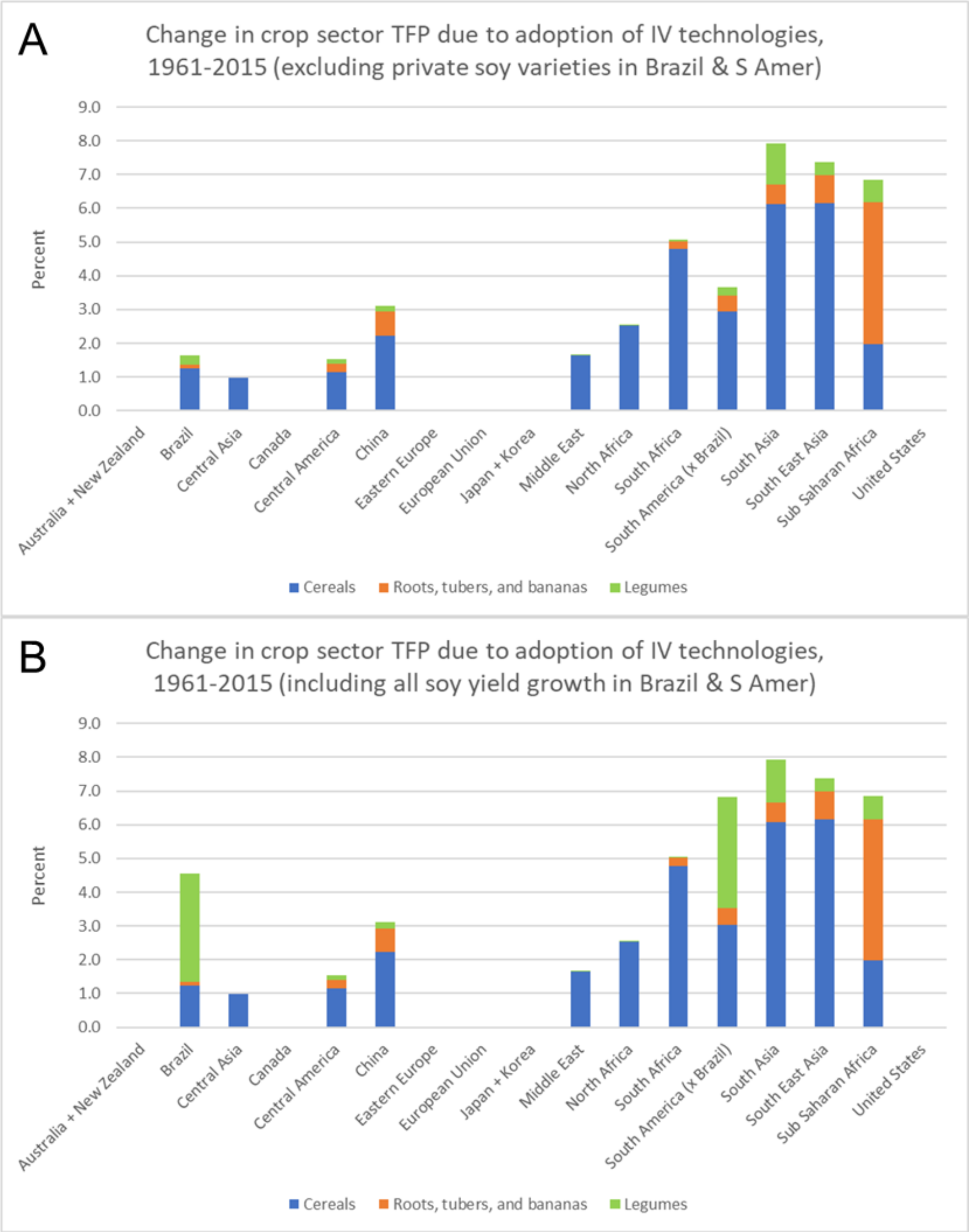

Fig. S11. Regional change in TFP over the period 1961 to 2015 by major crop groups

Notes: By 2015, private-sector GM varieties had largely replaced public soybean varieties in South America. In Fig. S11 B, TFP shocks assume that soybean IV adoption accounts for nearly all observed growth in average farm yields in South America over this period and does not consider potential labor-saving biases of GM traits.

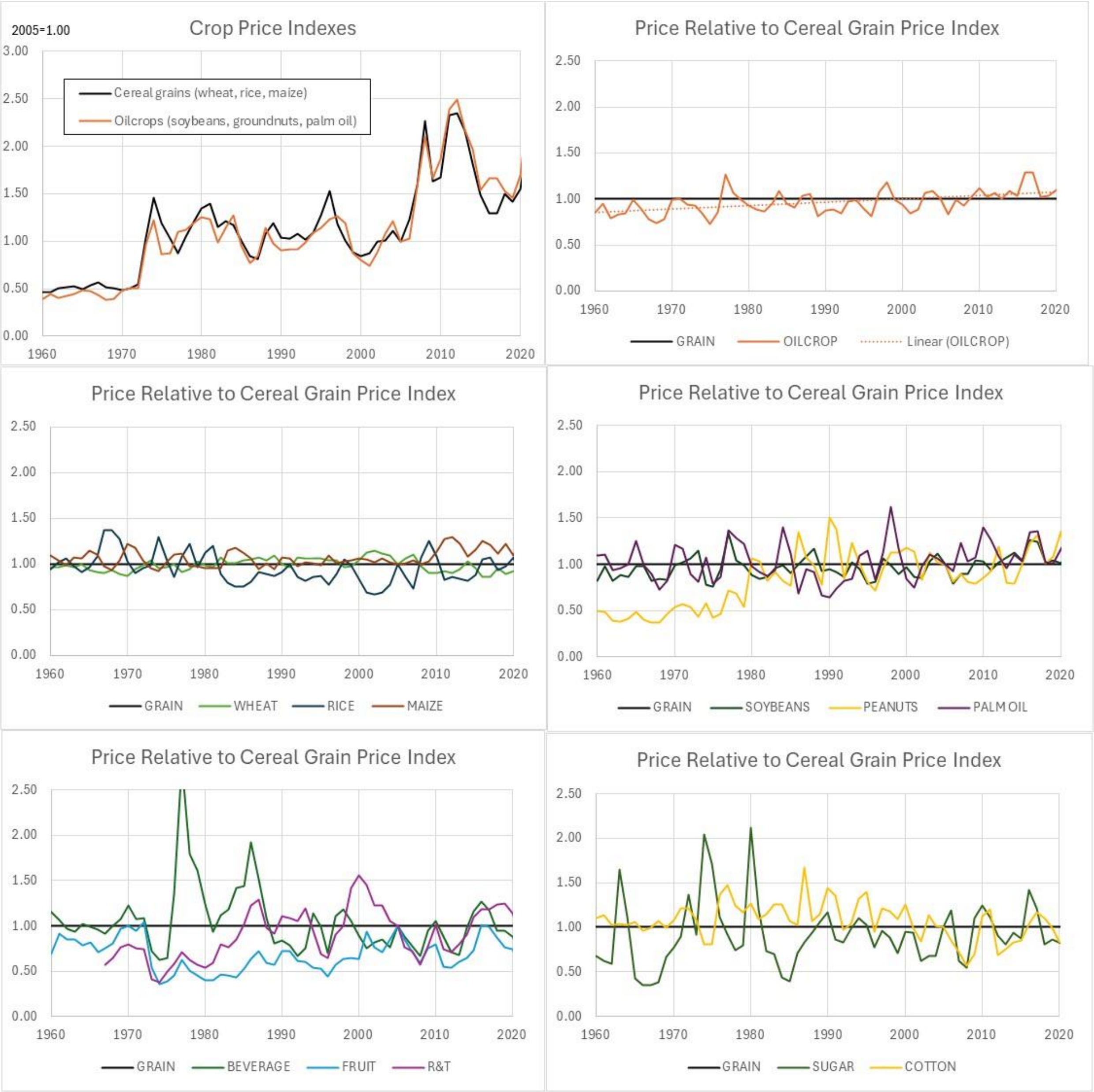

Fig. S12. Long-term co-movements among crop commodity prices

Source: Annual crop commodity prices series at major trading hubs are from IMF (28), except for root and tuber crops (tapioca starch export prices are from the Thailand Tapioca Starch Association (29) and U.S. wholesale frozen potato French fry prices are from the U.S. Federal Reserve(30).

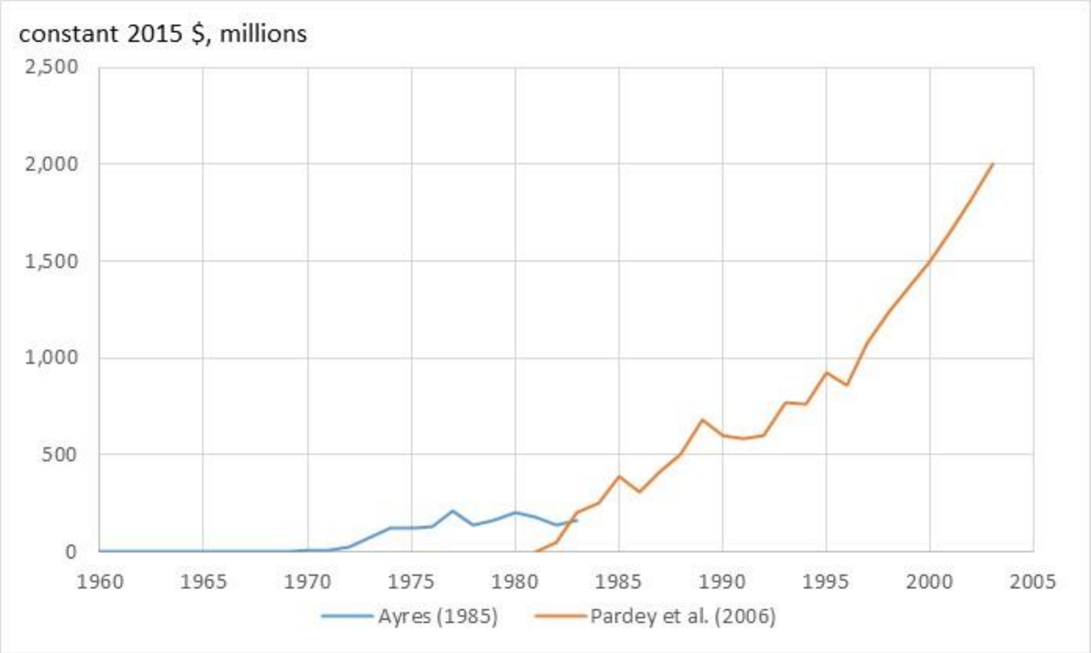

Fig. S13. Economic benefits from adoption of improved varieties of soybeans in Brazil

Sources: Ayres ((32), Table 4.4c) lists annual economic impacts from soybean variety adoption in constant 1977 Brazilian Cruzeiros (Cr$). Farmgate soybean prices in Brazil in 1977 are 3153.33 Cr$/T or 222.94 USD/T at the 1977 market exchange rate (37). The values are converted to 2015 PPP$ using the FAO global average soybean price of 382.46 PPP$/T (i.e., using a conversion factor of 0.1213 2015 PPP$ per 1.000 1977 Cr$); Pardey et al. ((33), Table 3) for annual values of economic benefits. These annual benefits were reported in constant 1999 US$ using a soybean price of 239.65 US$/T. They were converted to 2015 PPP$ using a conversion factor of 1.5959 2015 PPP$ per 1.000 1999 US$.

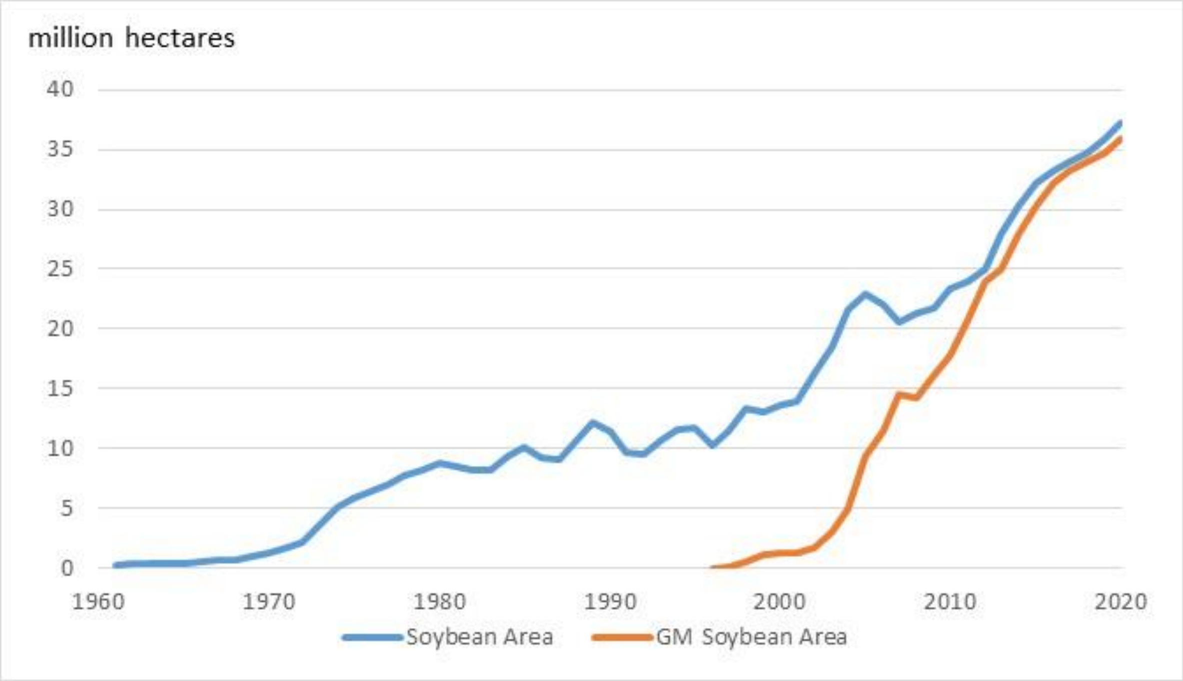

Fig. S14. Soybean area harvested and soybean area under GM soybean in Brazil, 1961-2020.

Source: Total soybean area harvested from FAOSTAT (9). Area under GM soybeans from ISAAA(38–51).

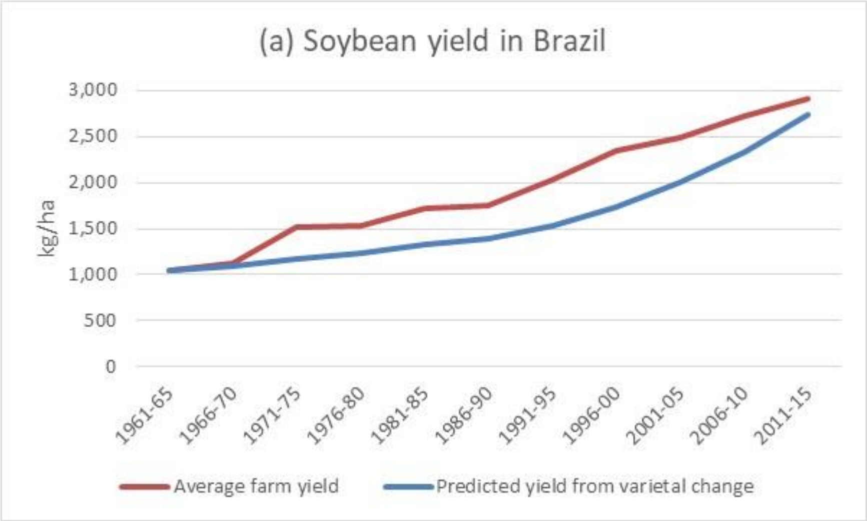

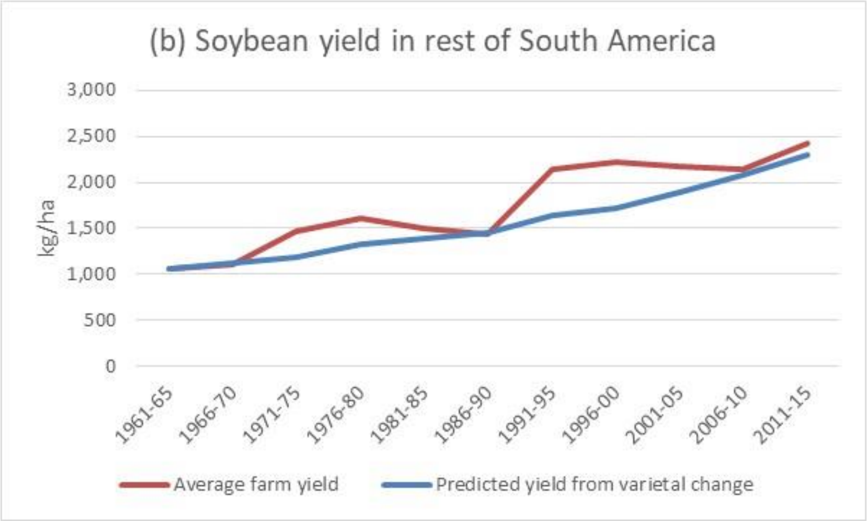

Fig. S15. Potential contributions of IV adoption to soybean yield growth in South America

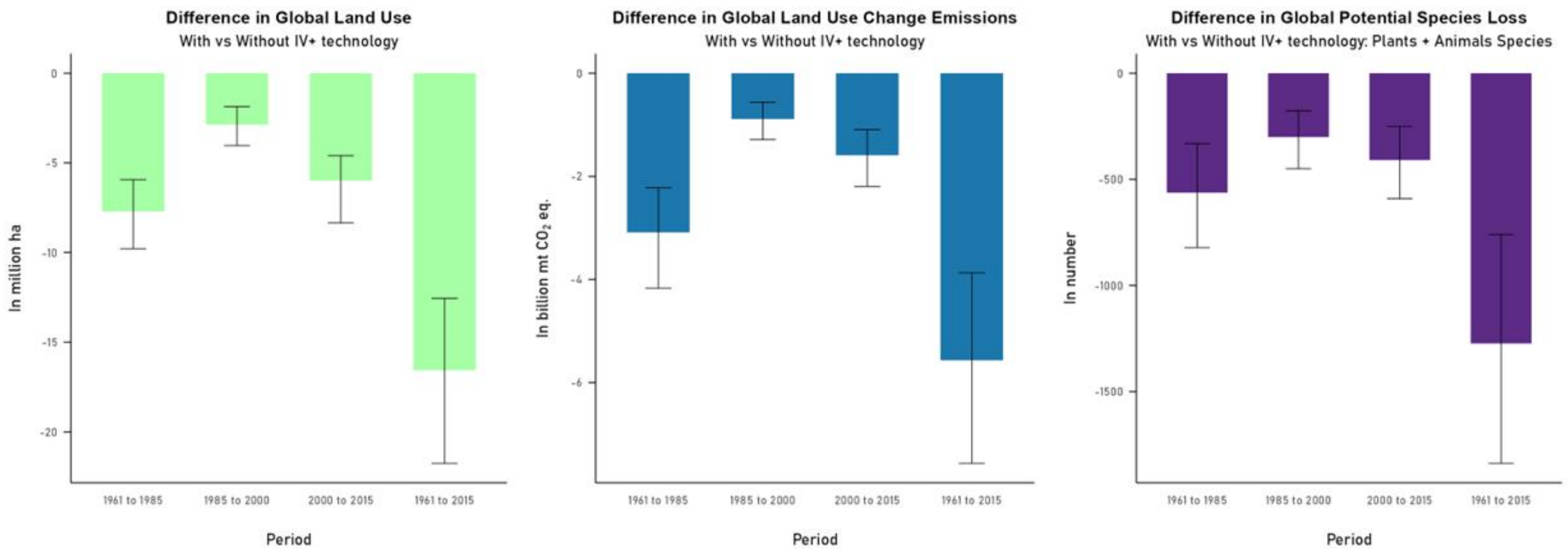

Fig. S16. Global changes in cropland use change, LUC GHG emissions and potential species loss due to IV adoption including potential contribution of soybean yield growth in South America for period 1961 to 2015. Error bars show 2.5% and 97.5% percentiles.

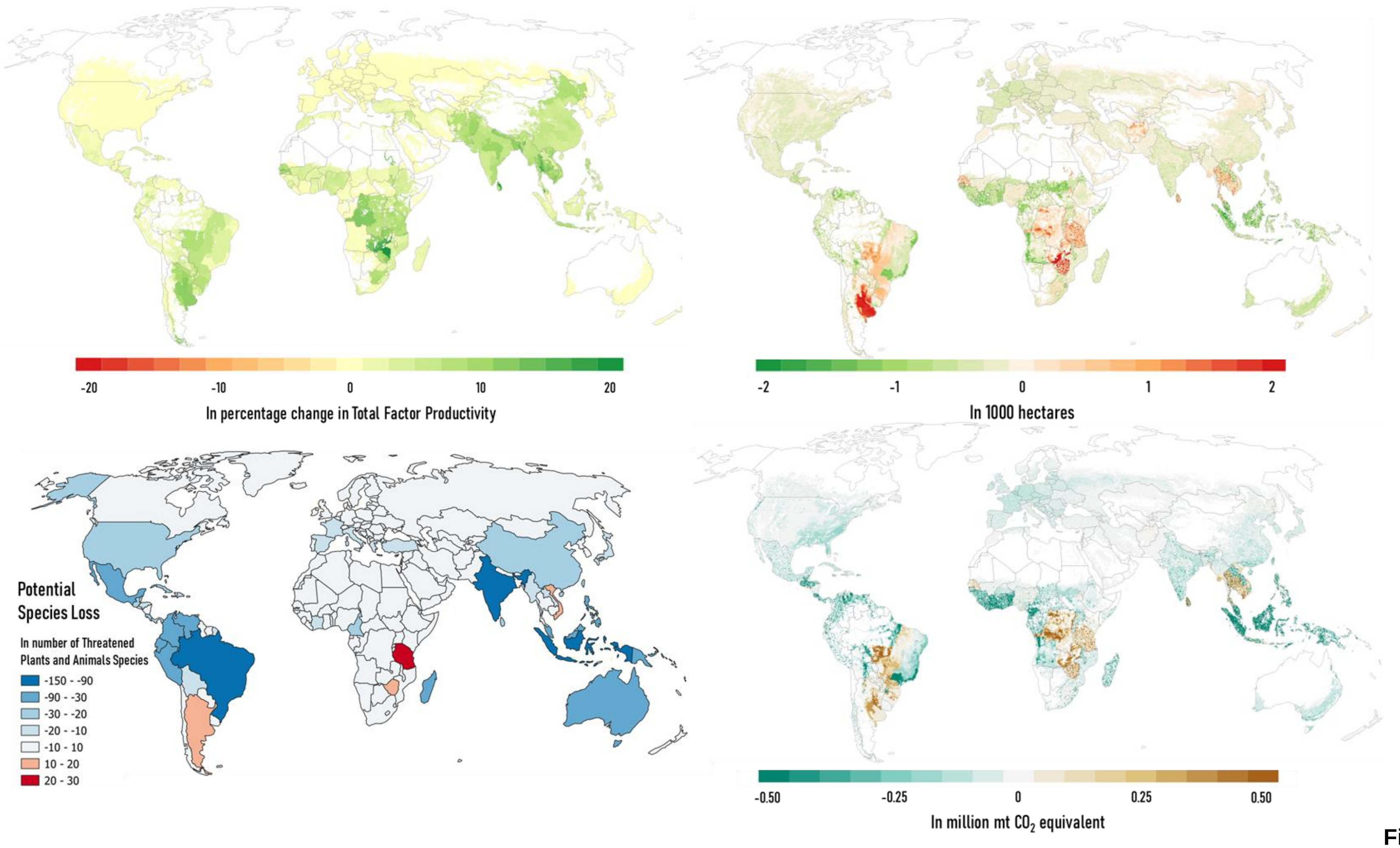

Fig. S17. Average changes in total factor productivity, cropland use, LUC GHG emissions and potential species loss due to IV adoption including potential contribution of soybean yield growth in South America for period 1961 to 2015.

Tab. S1. Key per annum growth rates used for the historical and counterfactual simulations

Regions	Population			Per Capital Income			Total Factor Productivity for Crops and Livestock			Total Factor Productivity for Proc. Food		
	1961-1985	1985-2000	2000-2015	1961-1985	1985-2000	2000-2015	1961-1985	1985-2000	2000-2015	1961-1985	1985-2000	2000-2015
Eastern Europe	0.78	0.00	-0.05	2.10	-4.24	4.24	-0.91	1.17	2.58	0.89	0.89	0.89
North Africa	2.51	2.06	1.65	0.94	0.37	1.94	0.92	2.19	2.21			
Sub Saharan Africa	2.65	2.80	2.81	0.92	-0.78	3.18	-0.45	1.20	0.92			
South America	2.19	1.81	1.19	1.05	1.34	2.37	1.15	1.34	1.77			
Brazil	2.47	1.70	1.03	4.09	0.51	2.38	1.17	1.84	3.23			
Australia / New Zealand	1.58	1.25	1.55	1.96	1.89	1.33	1.20	2.32	1.21			
European Union	0.53	0.27	0.39	2.64	2.09	0.80	0.92	1.41	1.79			
South Asia	2.31	2.09	1.50	1.37	3.21	4.72	0.75	1.03	2.68			
Central America	2.53	1.73	1.35	3.02	1.17	0.92	0.92	0.31	1.59			
South Africa	2.58	2.20	1.32	1.25	-0.34	1.94	-0.01	1.83	2.03			
South East Asia	2.52	1.84	1.30	3.49	3.68	3.73	1.13	1.63	2.30			
Canada	1.43	1.15	1.12	1.88	1.12	1.63	-0.23	2.54	1.36			
United States	0.95	1.05	0.88	2.30	2.11	0.86	1.21	1.74	1.52			
China	2.05	1.18	0.57	5.08	8.27	9.09	0.82	3.03	3.46			
Middle East	2.88	2.38	2.08	2.99	1.72	2.17	1.46	1.39	2.26			
Japan / Korea	1.30	0.48	0.14	4.50	2.29	1.03	1.39	1.42	1.23			
Central Asia	2.86	1.99	1.54	2.47	-3.05	6.02	-0.43	1.36	1.48			

Notes: Historical growth rates in regional population (9), per capita incomes (9), biofuels, TFP for crops and livestock (54) and TFP for processed food sector (55) are used to project the model from 1961 to 2015. Grid level changes in cropland cover (12) are also used to shift land supply in each grid cell in both historical baseline and counterfactual scenarios.

Tab. S2. Distribution of key model parameters used in the uncertainty analysis using Latin Hypercube Sampling (n=375)

Normal Distribution	Mean	Standard Deviation		Comments
Price Elasticities of Commodity Demand				Based on log linear regression of Per Capital Incomes and Elasticities estimated by Muhammed et al ((20))
Crops	-0.737	0.016		
Livestock	-0.826	0.013		
Processed Food	-1.172	0.019		
Income Elasticities of Commodity Demand				
Crops	0.882	0.024		
Livestock	1.048	0.019		
Processed Food	1.198	0.022		
**Triangular Distribution**	**Min**	**Max**	**Mode**	
Elasticities of Substitution in Production				+/- 30% from base parameter values
Crops	2.10	3.90	3.00	
Livestock	0.81	1.51	1.16	
Elasticities of Transformation in Crop Markets	2.10	3.90	3.00	
Elasticities of Substitution in Crop Markets	2.10	3.90	3.00	
Non-Land Input Supply Elasticities	0.94	1.74	1.34	
Cropland Supply Parameter	0.35	0.65	0.50	
Carbon Emission Factors	0.70	1.30	1.00	
Potential Species Loss Factors				Based on normalized global values from Chaudhary et al (16)
Mammals	0.63	1.59	1.00	
Birds	0.60	1.67	1.00	
Amphibians	0.63	1.60	1.00	
Reptiles	0.45	1.90	1.00	
Plants	0.34	2.44	1.00	

Notes: Global parameters which are scaled down to the local level using regional or grid-level scalar values.

Tab. S3. TFP shocks from adoption of improved varieties of food crops by 2016-2020

	Crop TFP shock by 2016-2020 (%)*								
	CROP SECTOR	CEREAL S	RTB	LEGUME S	OTHER CROPS	Wheat	Rice	Maize	Cassava
Sub-Saharan Africa	6.8	10.7	11.0	6.0	NA	15.7	7.4	15.5	27.1
Latin America	4.6	10.2	3.3	13.1	NA	35.0	6.3	7.6	3.3
Asia	4.4	15.1	12.7	16.8	NA	14.8	16.6	9.3	14.4
CWANA	1.8	10.0	NA	0.1	NA	15.1	3.0	NA	NA
All Regions	4.5	13.9	10.3	12.8	NA	16.5	15.7	9.3	21.3
	Crop value shares in 2016-2020								
	CROP SECTOR	CEREAL S	RTB	LEGUME S	OTHER CROPS	Wheat	Rice	Maize	Cassava
Sub-Saharan Africa	1.00	0.19	0.34	0.10	0.37	0.01	0.06	0.07	0.12
Latin America	1.00	0.20	0.08	0.17	0.56	0.02	0.04	0.12	0.01
Asia	1.00	0.22	0.05	0.02	0.71	0.03	0.14	0.04	0.01
CWANA	1.00	0.43	0.12	0.01	0.44	0.27	0.01	0.09	0.00
All Regions	1.00	0.21	0.08	0.05	0.67	0.04	0.11	0.05	0.02

*The crop TFP shock is the increase in a region's net crop production (value of gross crop production change minus changes in input costs) due to adoption of improved varieties.

CEREALS include wheat, rice, maize, barley, millet, and sorghum; RTB includes cassava, yam, potatoes, sweet potatoes, and bananas; LEGUMES include soybeans (only for Sub-Saharan Africa and Brazil), groundnuts, beans, cowpeas and four pulse crops (chickpea, lentils, fababean, and pigeon pea). OTHER CROPS include all other crops apart from these 19 food crops.

CWANA = Central & West Asia and North Africa. NA = TFP shock is not available and assumed to be zero.

Tab.S4. Simple correlations between crop commodity annual prices

	GRAINS	OILCROPS	BEVERAGE	FRUITS	R&T	WHEAT	RICE	MAIZE	SOYBEANS	PEANUTS	PALM OIL	COCOA	COFFEE-A	COFFEE-R	TEA	BANANAS	ORANGES	TAPIOCA	FRENCH FRIES	SUGAR	COTTON
CEREAL GRAINs	1.00																				
OILCROPS	0.97	1.00																			
BEVERAGES	0.73	0.78	1.00																		
FRUITS	0.83	0.86	0.59	1.00																	
ROOT & TUBERS	0.75	0.80	0.49	0.86	1.00																
WHEAT	0.99	0.94	0.70	0.80	0.73	1.00															
RICE	0.91	0.88	0.69	0.74	0.55	0.85	1.00														
MAIZE	0.98	0.98	0.71	0.84	0.78	0.95	0.85	1.00													
SOYBEANS	0.97	0.99	0.77	0.87	0.80	0.94	0.87	0.97	1.00												
PEANUTS	0.88	0.90	0.67	0.83	0.86	0.86	0.80	0.88	0.89	1.00											
PALM OIL	0.94	0.97	0.77	0.80	0.71	0.91	0.85	0.95	0.94	0.82	1.00										
COCOA	0.72	0.77	0.96	0.63	0.51	0.69	0.72	0.69	0.77	0.66	0.74	1.00									
COFFEE-Arabica	0.76	0.81	0.95	0.62	0.56	0.74	0.67	0.76	0.79	0.72	0.80	0.86	1.00								
COFFEE-Robusta	0.48	0.50	0.89	0.26	0.11	0.47	0.46	0.45	0.50	0.42	0.50	0.80	0.82	1.00							
TEA	0.74	0.81	0.78	0.73	0.76	0.69	0.69	0.76	0.78	0.81	0.78	0.77	0.77	**0.54**	1.00						
BANANAS	0.76	0.82	0.36	0.93	0.93	0.70	0.65	0.79	0.83	0.80	0.74	0.39	0.44	-0.11	0.64	1.00					
ORANGES	0.73	0.76	0.51	0.94	0.67	0.71	0.68	0.72	0.76	0.69	0.71	0.57	0.51	0.22	0.61	0.73	1.00				
TAPIOCA	0.83	0.87	0.88	0.80	0.83	0.74	0.78	0.85	0.86	0.75	0.83	0.82	0.81	0.70	0.66	0.88	0.57	1.00			
FRENCH FRIES	0.77	0.83	0.51	0.93	0.97	0.73	0.61	0.80	0.83	0.85	0.75	0.53	0.58	0.12	0.75	0.97	0.75	0.87	1.00		
SUGAR	0.79	0.75	0.58	0.63	0.46	0.75	0.83	0.75	0.74	0.70	0.72	0.58	0.60	0.40	0.55	0.55	0.57	0.80	0.50	1.00	
COTTON	0.84	0.84	0.79	0.64	0.62	0.84	0.73	0.82	0.82	0.80	0.81	0.73	0.84	0.63	0.72	0.48	0.52	0.75	0.59	0.74	1.00

* Price are average annual price indexes (2005=1.00) from 1957 to 2023, except for potato frozen french fries which begins in 1967, bananas which begins in 1975, and tapioca which begins in 1990.

Cereal grains include wheat, rice and maize; Oilcrops include soybeans, peanuts and palm oil; Beveral crops include cocoa, coffee and tea; Fruit crops include bananas and oranges; Root & Tuber crops include cassava starch (tapicoa)and potato frozen french fries. The sugar price is CSCE free market price.

Source of crop price data: International Monetary Fund (IMF) Commodity Price Database, except Thai tapioca starch export price (Thailand Tapioca Association) and U.S. wholesale price for frozen French fries (U.S. Federal Reserve). Prices are international trade prices at major import/export hubs, except the potato French fry price.

Tab.S5. Adoption of Improved Varieties and by 2016–2020

Region	All listed crops	Improved Varieties (1000 ha)															
		Wheat	Rice	Maize	Sorghum	Millet	Barley	Cassava	Potato	Yam	Sweet potato	Banana	Beans	Cowpea	Groundnut	Soybean	Pulses
China	110,278	24,112	30,561	42,280	576	-	-	294	4,537	-	2,323	-	745	-	3,938	-	912
SE Asia	52,859	-	39,373	8,169	63	-	-	2,602	104	-	175	-	-	-	1,467	-	905
India	121,647	30,246	43,892	8,156	4,212	6,073	513	134	2,003	-	80	-	7,002	-	3,459		15,877
Other South Asia	28,706	9,761	14,314	2,496	-	-	-	-	751	-	1	-	-	-	19	-	1,364
**ASIA**	**313,491**	**64,119**	**128,140**	**61,101**	**4,852**	**6,073**	**513**	**3,030**	**7,395**	**-**	**2,579**	**-**	**7,748**	**-**	**8,883**	**-**	**19,058**
Argentina	12,854	5,624	-	6,728	-	-	-	-	-	-	-	-	399	-	-	-	102
Brazil	21,912	2,138	921	16,690	-	-	-	126	115	-	-	-	1,893	-	-	-	29
Other South America	4,735	1,228	791	1,883	-	-	9	44	524	-	4	-	144	-	-	-	109
Cent. America & Carib.	8,260	615	286	6,330	-	-	-	207	79	-	-	-	505	-	-	-	237
**LAC**	**47,761**	**9,605**	**1,998**	**31,631**	**-**	**-**	**9**	**376**	**718**	**-**	**4**	**-**	**2,941**	**-**	**-**	**-**	**478**
Nigeria	17,159	57	2,726	3,377	1,099	471	-	4,576	-	1,570	-	-	-	1,795	728	759	-
Other West & Cent. Africa	19,528	-	2,628	5,256	1,747	1,786	-	3,247	22	1,022	-	-	68	1,752	848	246	905
South & East Africa	27,946	2,680	769	14,507	3,990	188	405	1,426	306	-	91	179	1,504	95	1,328	463	14
**SSA**	**65,275**	**2,737**	**6,123**	**23,140**	**6,835**	**2,445**	**405**	**9,250**	**328**	**2,592**	**91**	**179**	**1,573**	**3,643**	**2,905**	**1,468**	**1,561**
North Africa	8,278	6,596	258	-	68	-	943	-	-	-	-	-	-	-	-	-	413
West Asia	17,906	15,817	-	-	-	-	484	-	-	-	-	-	-	-	-	-	1,605
Central Asia	3,435	3,427	-	-	-	-	-	-	-	-	-	-	-	-	-	-	9
**CWANA**	**29,620**	**25,839**	**258**	**-**	**68**	**-**	**1,427**	**-**	**-**	**-**	**-**	**-**	**-**	**-**	**-**	**-**	**2,027**
**ALL DEVELOPING COUNTRIES**	**456,146**	**102,300**	**136,520**	**115,872**	**11,755**	**8,517**	**2,354**	**12,656**	**8,441**	**2,592**	**2,674**	**179**	**12,261**	**3,643**	**11,788**	**1,468**	**23,125**

Source: Fuglie and Echeverria (27)

Tab.S6. Share of Total Crop Area in Improved Varieties by 2016–2020

Region	All listed crops	Improved Varieties (percent of crop area)															
		Wheat	Rice	Maize	Sorghum	Millet	Barley	Cassava	Potato	Yam	Sweet potato	Banana	Beans	Cowpea	Groundnut	Soybean	Pulses
China	96	99	99	99	91			99	96		97		66		86		0
SE Asia	78		88	79	31			79	75		41				84		0
India	86	100	100	85	80	67	83	74	93	-	66	-	50	-	66		11
Other South Asia	85	98	85	90					87		5				12		8
**ASIA**	**87**	**99**	**94**	**93**	**77**	**56**	**47**	**80**	**94**		**86**		**40**		**76**		**10**
Argentina	84	100		99					98				95				-
Brazil	83	100	50	99				10	96				70				-
Other South America	48	95	44	67			2	10	62		12		40				-
Cent. America & Carib.	44	100	28	63				54	61				18				-
**LAC**	**68**	**99**	**41**	**87**			**1**	**18**	**69**		**2**		**47**				**-**
Nigeria	38	99	50	47	20	25		66		25				39	19	89	
Other West & Cent. Africa	31		33	50	11			38	39	46			5	65		68	0
South & East Africa	32	93	30	64	26	11	35	26	34		4	6	22	17	22	35	11
**SSA**	**33**	**93**	**38**	**58**	**24**	**12**	**35**	**44**	**25**	**30**	**2**	**3**	**19**	**26**	**18**	**58**	**11**
North Africa	60	95	49		43		29										0
West Asia	55	89					7										2
Central Asia	53	69															-
**CWANA**	**56**	**87**	**17**		**13**		**13**										**1**
**ALL DEVELOPING COUNTRIES**	**67**	**96**	**86**	**80**	**31**	**28**	**16**	**47**	**74**	**30**	**36**	**2**	**36**	**25**	**41**	**2**	**9**

Source: Fuglie and Echeverria (27)

References

1. T. W. Hertel, The Global Supply and Demand for Agricultural Land in 2050: A Perfect Storm in the Making? *American Journal of Agricultural Economics* **93**, 259 (2011).

2. S. Zuidema, *et al.*, US climate policy yields water quality cobenefits in the Mississippi Basin and Gulf of Mexico. *Proceedings of the National Academy of Sciences* **120**, e2302087120 (2023).

3. J. Liu, *et al.*, Tackling policy leakage and targeting hotspots could be key to addressing the" Wicked" challenge of nutrient pollution from corn production in the US. *Environmental Research Letters* (2023).

4. Z. Wang, G. B. Martha, J. Liu, C. Z. Lima, T. W. Hertel, Planned expansion of transportation infrastructure in Brazil has implications for the pattern of agricultural production and carbon emissions. *Science of The Total Environment* **928**, 172434 (2024).

5. I. Haqiqi, *et al.*, Local, regional, and global adaptations to a compound pandemic-weather stress event. *Environ. Res. Lett.* **18**, 035005 (2023).

6. K. Fuglie, S. Ray, U. L. C. Baldos, T. W. Hertel, The R&D cost of climate mitigation in agriculture. *Applied Economic Perspectives and Policy* **44**, 1955–1974 (2022).

7. D. McFadden, Constant Elasticity of Substitution Production Functions. *The Review of Economic Studies* **30**, 73–83 (1963).

8. T. W. Hertel, J. Mark Horridge, K. R. Pearson, Mending the family tree a reconciliation of the linearization and levels schools of AGE modelling. *Economic Modelling* **9**, 385–407 (1992).

9. FAO, FAOSTAT Database. (2023). Available at: http://faostat.fao.org/ [Accessed 25 September 2023].

10. C. Monfreda, N. Ramankutty, J. A. Foley, Farming the planet: 2. Geographic distribution of crop areas, yields, physiological types, and net primary production in the year 2000. *Global Biogeochem. Cycles* **22**, 19 PP. (2008).

11. Global ADMinistrative Areas (GADM), Global Administrative Areas. (2016). Available at: http://gadm.org/country [Accessed 2 February 2017].

12. G. C. Hurtt, *et al.*, Harmonization of global land use change and management for the period 850–2100 (LUH2) for CMIP6. *Geoscientific Model Development* **13**, 5425–5464 (2020).

13. A. Aguiar, M. Chepeliev, E. L. Corong, R. McDougall, D. van der Mensbrugghe, The GTAP Data Base: Version 10. *Journal of Global Economic Analysis* **4**, 1–27 (2019).

14. F. Taheripour, L. Pena-Levano, W. Tyner, "Introducing first and second generation biofuels into GTAP 9 Data Base" (Global Trade Analysis Project (GTAP), 2017).

15. P. C. West, *et al.*, Trading carbon for food: Global comparison of carbon stocks vs. crop yields on agricultural land. *PNAS* (2010). https://doi.org/10.1073/pnas.1011078107.

16. A. Chaudhary, F. Verones, L. de Baan, S. Hellweg, Quantifying Land Use Impacts on Biodiversity: Combining Species–Area Models and Vulnerability Indicators. *Environ. Sci. Technol.* **49**, 9987–9995 (2015).

17. B. Eickhout, H. van Meijl, A. A. Tabeau, E. Stehfest, "The impact of environmental and climate constraints on global food supply" in *Economic Analysis of Land Use in Global Climate Change Policy*, T. Hertel, S. Rose, R. Tol, Eds. (Routledge, 2009).

18. G. Woltjer, *et al.*, The MAGNET Model: Module description. (2014).

19. J. M. Schneider, F. Zabel, W. Mauser, Global inventory of suitable, cultivable and available cropland under different scenarios and policies. *Sci Data* **9**, 527 (2022).

20. A. Muhammad, J. L. Seale Jr., B. Meade, A. Regmi, "International Evidence on Food Consumption Patterns: An Update Using 2005 International Comparison Program Data" (Economic Research Service, US Department of Agriculture, 2011).

21. R. Keeney, T. W. Hertel, Indirect Land use Impacts of US Biofuels Policies: The Importance of Acreage, Yield and Bilateral Trade Responses. *American Journal of Agricultural Economics* **91**, 895–909 (2009).

22. U. L. Baldos, T. Hertel, SIMPLE: a Simplified International Model of agricultural Prices, Land use and the Environment. *GTAP Working Paper No. 70* (2012). Available at: http://www.gtap.agecon.purdue.edu/resources/res_display.asp?RecordID=4021.

23. R. Keeney, T. W. Hertel, "GTAP-AGR: A Framework for Assessing the Implications of Multilateral Changes in Agricultural Policies" (Center for Global Trade Analysis, Department of Agricultural Economics, Purdue University, 2005).

24. S. A. Ahmed, T. W. Hertel, R. Lubowski, "Calibration of a Land Cover Supply Function Using Transition Probabilities" (Center for Global Trade Analysis, Department of Agricultural Economics, Purdue University, 2008).

25. J. R. Stevenson, N. Villoria, D. Byerlee, T. Kelley, M. Maredia, Green Revolution research saved an estimated 18 to 27 million hectares from being brought into agricultural production. *PNAS* **110**, 8363–8368 (2013).

26. T. W. Hertel, N. Ramankutty, U. L. C. Baldos, Global market integration increases likelihood that a future African Green Revolution could increase crop land use and

CO2 emissions. *Proceedings of the National Academy of Sciences* **111**, 13799–13804 (2014).

27. K. O. Fuglie, R. G. Echeverria, The economic impact of CGIAR-related crop technologies on agricultural productivity in developing countries, 1961–2020. *World Development* **176**, 106523 (2024).

28. International Monetary Fund, IMF Primary Commodity Prices Database.

29. Thai Tapioca Starch Association, Statistics: Weekly Tapioca Starch Price.

30. Federal Reserve Bank of Minneapolis, Consumer Price Index and Inflation Rates (Estimate), 1800-. (2014).

31. F. Pendrill, *et al.*, Agricultural and forestry trade drives large share of tropical deforestation emissions. *Global Environmental Change* **56**, 1–10 (2019).

32. C. H. S. Ayres, The contribution of agricultural research to soybean productivity in Brazil. (1985).

33. P. G. Pardey, J. M. Alston, C. Chan-Kang, E. C. Magalhães, S. A. Vosti, International and Institutional R&D Spillovers: Attribution of Benefits among Sources for Brazil's New Crop Varieties. *American Journal of Agricultural Economics* **88**, 104–123 (2006).

34. P. Bustos, B. Caprettini, J. Ponticelli, Agricultural Productivity and Structural Transformation: Evidence from Brazil. *American Economic Review* **106**, 1320–1365 (2016).

35. V. Zalles, *et al.*, Near doubling of Brazil's intensive row crop area since 2000. *Proceedings of the National Academy of Sciences* **116**, 428–435 (2019).

36. E. J. Trigo, E. J. Cap, Ten years of genetically modified crops in Argentine agriculture. (2006).

37. "World Development Indicators" (World Bank, 2023).

38. ISAAA, "Global Status of Commercialized Biotech/GM Crops" (International Service for the Acquisition of Agri-biotech Applications, 2016).

39. ISAAA, "Global Status of Commercialized Biotech/GM Crops" (International Service for the Acquisition of Agri-biotech Applications, 2018).

40. ISAAA, "Global Status of Commercialized Biotech/GM Crops" (International Service for the Acquisition of Agri-biotech Applications, 2019).

41. ISAAA, "Global Status of Commercialized Biotech/GM Crops" (International Service for the Acquisition of Agri-biotech Applications, 2017).

42. ISAAA, “Global Status of Commercialized Biotech/GM Crops” (International Service for the Acquisition of Agri-biotech Applications, 2014).

43. ISAAA, “Global Status of Commercialized Biotech/GM Crops” (International Service for the Acquisition of Agri-biotech Applications, 2013).

44. ISAAA, “Global Status of Commercialized Biotech/GM Crops” (International Service for the Acquisition of Agri-biotech Applications, 2012).

45. ISAAA, “Global Status of Commercialized Biotech/GM Crops” (International Service for the Acquisition of Agri-biotech Applications, 2011).

46. ISAAA, “Global Status of Commercialized Biotech/GM Crops” (International Service for the Acquisition of Agri-biotech Applications, 2010).

47. ISAAA, “Global Status of Commercialized Biotech/GM Crops” (International Service for the Acquisition of Agri-biotech Applications, 2009).

48. ISAAA, “Global Status of Commercialized Biotech/GM Crops” (International Service for the Acquisition of Agri-biotech Applications, 2008).

49. ISAAA, “Global Status of Commercialized Biotech/GM Crops” (International Service for the Acquisition of Agri-biotech Applications, 2007).

50. ISAAA, “Global Status of Commercialized Biotech/GM Crops” (International Service for the Acquisition of Agri-biotech Applications, 2006).

51. ISAAA, “Global Status of Commercialized Biotech/GM Crops” (International Service for the Acquisition of Agri-biotech Applications, 2005).

52. R. A. Mittermeier, W. R. Turner, F. W. Larsen, T. M. Brooks, C. Gascon, “Global Biodiversity Conservation: The Critical Role of Hotspots” in *Biodiversity Hotspots: Distribution and Protection of Conservation Priority Areas*, F. E. Zachos, J. C. Habel, Eds. (Springer, 2011), pp. 3–22.

53. M. Hoffman, K. Koenig, G. Bunting, J. Costanza, K. J. Williams, Biodiversity Hotspots (version 2016.1). Zenodo. https://doi.org/10.5281/zenodo.3261807. Deposited 25 April 2016.

54. USDA ERS, International Agricultural Productivity. (2024). Available at: https://www.ers.usda.gov/data-products/international-agricultural-productivity/ [Accessed 24 July 2023].

55. R. Griffith, S. Redding, J. V. Reenen, Mapping the Two Faces of R&D: Productivity Growth in a Panel of OECD Industries. *Review of Economics and Statistics* **86**, 883–895 (2004).

56. Nuthalapati, C.S., A. Kumar, P.S. Birthal, V.K. Sonkar, Demand-side and Supply-side Factors for Accelerating Varietal Turnover in Smallholder Soybean Farms. Journal of Cleaner Production 447, 141372 (2024).
